# Synthesis and Application of a Chitosan-Based AM/AMPS Flocculant for the Flocculation of Kaolinite Suspensions: Experimental and Molecular Dynamics Study

**DOI:** 10.3390/ma19214701

**Published:** 2026-11-04

**Authors:** Jianbing Lv, Xunzan Peng, Dingyang Wu, Xiaodong Wan, Fangdong Fan, Chen Liang

**Affiliations:** 1 School of Civil and Transportation Engineering, Guangdong University of Technology, Guangzhou 510006, China; 2 Shenzhen Road and Bridge Construction Group Co., Ltd., Shenzhen 518029, China

**Keywords:** chitosan-based flocculant, kaolinite suspensions, graft copolymerization, flocculation, molecular dynamics simulations

## Abstract

Fine kaolinite particles form stable suspensions that impede solid–liquid separation. A chitosan-based flocculant, CS-g-P(AM-AMPS), was synthesized by ultrasound-assisted graft copolymerization of acrylamide (AM) and 2-acrylamido-2-methylpropanesulfonic acid (AMPS) onto chitosan (CS). Physicochemical characterization, batch flocculation tests, and molecular dynamics simulations were conducted with the AM/AMPS copolymer P(AM-AMPS) and polyacrylamide (PAM) as reference polymers. Characterization identified amide and sulfonate groups and a rough, porous dry-state morphology. CS-g-P(AM-AMPS) achieved higher final turbidity removal and produced larger median flocs than both reference polymers under their respective selected conditions, with a maximum removal efficiency of 98.5%. Simulations showed that the grafted polymer had the most negative total polymer-surface interaction energy and the largest interfacial hydrogen-bond population. Its segment distribution combined near-surface enrichment with extension into the adjacent aqueous phase. P(AM-AMPS) produced larger median flocs than PAM but achieved lower removal, showing that aggregate size and clarification followed different rankings. These findings suggest that extensive surface contact coupled with chain extension facilitates particle bridging and floc growth. The combined results identify CS-g-P(AM-AMPS) as an effective flocculant for clarifying kaolinite-bearing water.

## 1. Introduction

Fine-grained clay minerals, particularly kaolinite, are common in groundwater aquifers, engineering slurries, tailings, and other clay-bearing porous media. Their small particle size, platy morphology, surface charge, and strong hydration promote stable colloidal suspensions that settle slowly. Once mobilized by flowing water, these particles can migrate through pore channels and accumulate at constrictions, progressively reducing permeability [1,2,3]. Controlled aggregation and removal of suspended kaolinite are therefore relevant to both water clarification and the management of particle-induced physical clogging. Chitosan-based materials have been used in applications ranging from hydrogels to clay stabilization [4,5], indicating that the chemical and structural versatility of chitosan may also be useful in the design of mineral-particle flocculants.

Coagulation and polymer flocculation are established methods for destabilizing suspended particles and promoting solid–liquid separation [6,7,8]. Among bio-based polymers, chitosan (CS) is of particular interest because its amino groups can be protonated under acidic conditions, while its amino and hydroxyl groups provide potential interaction sites for negatively charged particles and mineral surfaces [9,10,11]. Polyacrylamide (PAM), in contrast, provides flexible, high-molecular-weight chains that can capture and bridge particles, although its performance depends strongly on molecular-weight distribution and chain conformation [12]. The compatibility of chitosan with mineral phases has also supported its use in geotechnical stabilization and carbonate-precipitation-based repair systems [5,13]. Graft polymerization and composite formation have consequently been explored to improve chitosan solubility, chain extension, and the accessibility of binding sites [14,15,16]. Studies of charged and amphoteric biopolymer flocculants further indicate that functional-group composition and polymer architecture influence particle destabilization and floc growth [17,18].

Chitosan-mineral composites and ultrasound-assisted polymer synthesis offer additional routes for tuning material composition and synthesis efficiency [19,20]. These studies establish useful relationships between polymer chemistry, architecture, and treatment performance. Most assessments, however, rely primarily on bulk or endpoint measurements and therefore provide limited information on the molecular events that precede aggregation. In particular, the roles of surface association, polymer conformation, molecular mobility, and interfacial hydration are difficult to distinguish from removal efficiency or settling behavior alone.

This limitation is especially relevant to graft copolymers, whose backbones and side chains may contribute differently to initial surface attachment and subsequent interparticle bridging. Measurements of turbidity, settling behavior, particle size, and morphology describe the overall aggregation response but do not distinguish these contributions. Wang et al. prepared CTS-g-P(AM-DMC) by plasma-initiated graft copolymerization of acrylamide and [2-(methacryloyloxy)ethyl]trimethylammonium chloride (DMC) chloride onto chitosan. They evaluated the effects of graft ratio, dosage, and pH on kaolin flocculation, achieving 98.6% turbidity removal at 0.6 mg/L and pH 4. The study showed that charge neutralization predominated under acidic conditions, whereas electrostatic patching played a greater role under neutral conditions [21]. These results linked graft composition to clarification performance but left the organization of adsorbed chains unresolved. Gurina et al. used MD simulations to examine poly(N-vinyl-2-pyrrolidone) adsorption on hydrophilic and hydrophobic cellulose nanocrystal surfaces in vacuum and water. Their analyses of chain conformation, hydrogen bonding, and interaction energies showed that water weakened polymer-cellulose interactions and that adsorption in water involved globular chains interacting mainly through solvation shells [22]. This work established a molecular connection between hydration and polymer adsorption. How chitosan-based AM/AMPS graft architecture influences surface association and chain extension at hydrated kaolinite interfaces, and how these features relate to floc growth and clarification, remains to be clarified.

Molecular dynamics (MD) simulation provides a useful means of examining polymer adsorption and conformational rearrangement in hydrated solid–liquid systems, where the relevant interfacial processes are difficult to isolate experimentally [22]. Cationic polyacrylamide (CPAM) is a polyacrylamide-based flocculant containing positively charged functional groups. Studies of CPAM structural units and chitosan-g-PAM have identified electrostatic attraction and hydrogen bonding as important adsorption interactions and shown that polymer conformation varies with mineral surface chemistry and solution salinity [23,24]. Studies combining experimental measurements with MD simulations have further related polymer adsorption and interfacial configuration to the separation behavior of clay-containing suspensions [25]. Xu et al. combined MD calculations of representative polyacrylamide-based polymers with dissolution tests of AM/AMPS copolymers, relating functional-group polarity, charge density, chain flexibility, and hydration to polymer configuration and solution behavior [26]. In a particulate suspension, Lu et al. associated PAM adsorption, bridging, and chain entanglement with changes in macroscopic slurry behavior [27]. In the present study, experimental tests establish differences in kaolinite removal and floc formation, while MD simulations relate these differences to polymer adsorption and chain configuration at the kaolinite/water interface.

Accordingly, the present study examines whether incorporating a CS backbone into P(AM-AMPS) is associated with changes in kaolinite removal and floc development, and which interfacial processes may account for these changes. CS-g-P(AM-AMPS) is compared with the corresponding ungrafted P(AM-AMPS) and with linear PAM under a common experimental and simulation framework. P(AM-AMPS) provides a reference for evaluating the contribution of the chitosan-containing graft architecture within the AM/AMPS system, while PAM serves as a conventional linear-polymer benchmark.

CS-g-P(AM-AMPS) was synthesized by ultrasound-assisted free-radical graft copolymerization and characterized in terms of chemical composition, thermal behavior, and surface morphology. Batch experiments evaluated the effects of polymer dosage, solution pH, and flocculation time on kaolinite removal, followed by particle-size analysis under the respective optimum conditions. In parallel, hydrated interfacial models were constructed for CS-g-P(AM-AMPS), P(AM-AMPS), and PAM on the aluminol basal face of kaolinite (001). The simulations compared polymer–kaolinite interaction energies, radii of gyration, mean-square displacements, diffusion coefficients, relative concentration profiles, and interfacial hydrogen-bond populations.

Together, the experimental and simulation analyses relate kaolinite removal and floc growth to polymer adsorption and chain configuration at the hydrated mineral interface. They clarify the coupled roles of chitosan-backbone anchoring and AM/AMPS graft-chain extension, providing a molecular basis for the further development of chitosan-based flocculants for fine-particle separation.

## 2. Materials and Methods

### 2.1. Materials and Polymer Preparation

CS, AMPS, anhydrous ethanol, and AM used in the experiment were all purchased from Wuhan Sinopharm Chemical Reagent Co., Ltd. (Shanghai, China). The pH of the reaction solution was adjusted using 0.1 mol/L HCl and NaOH solutions, measured with a pH meter (S40, Mettler-Toledo, Switzerland).

**Figure 1:**
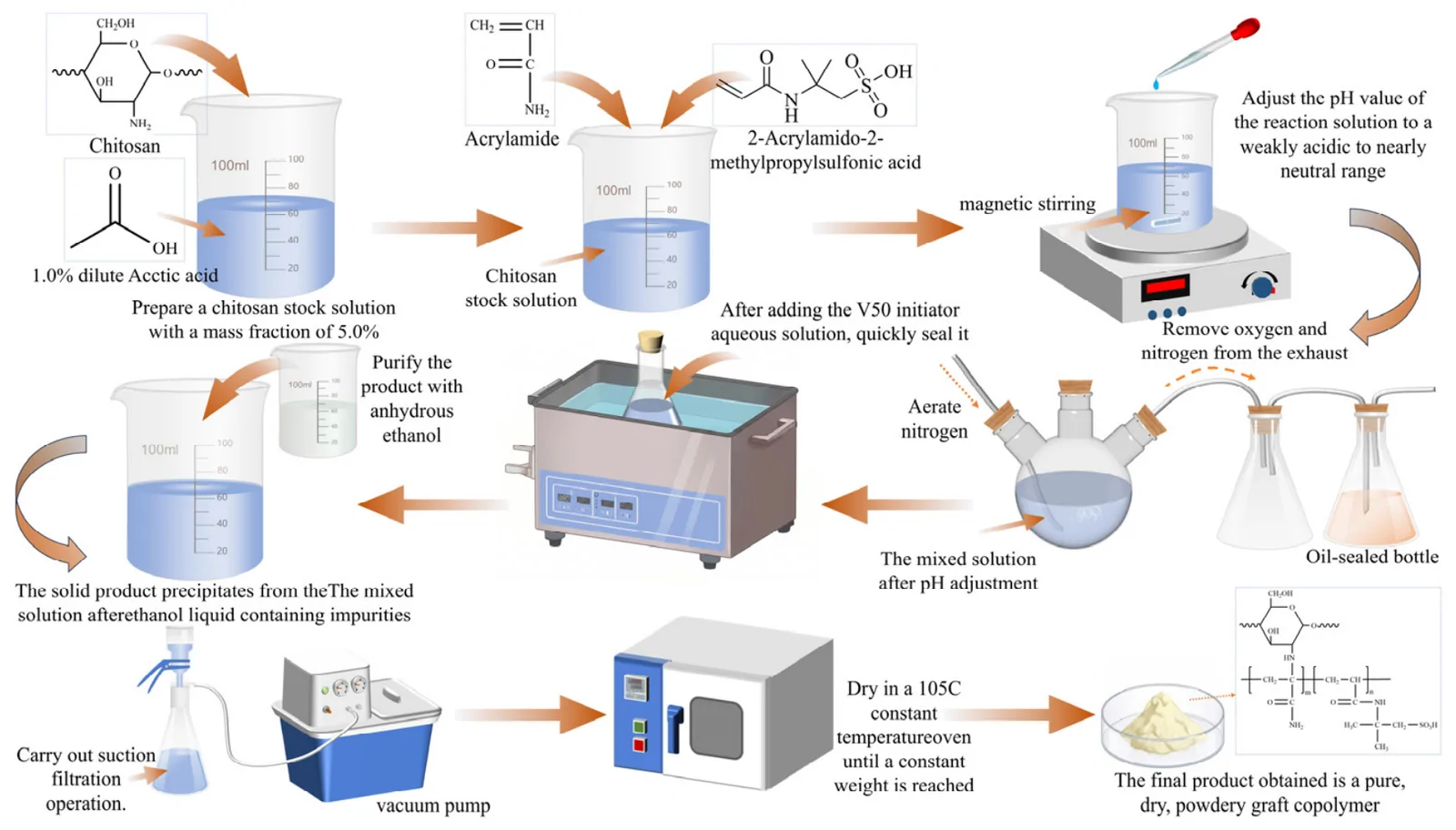
Synthetic route and experimental workflow of CS-g-P(AM-AMPS).

**Figure 2:**
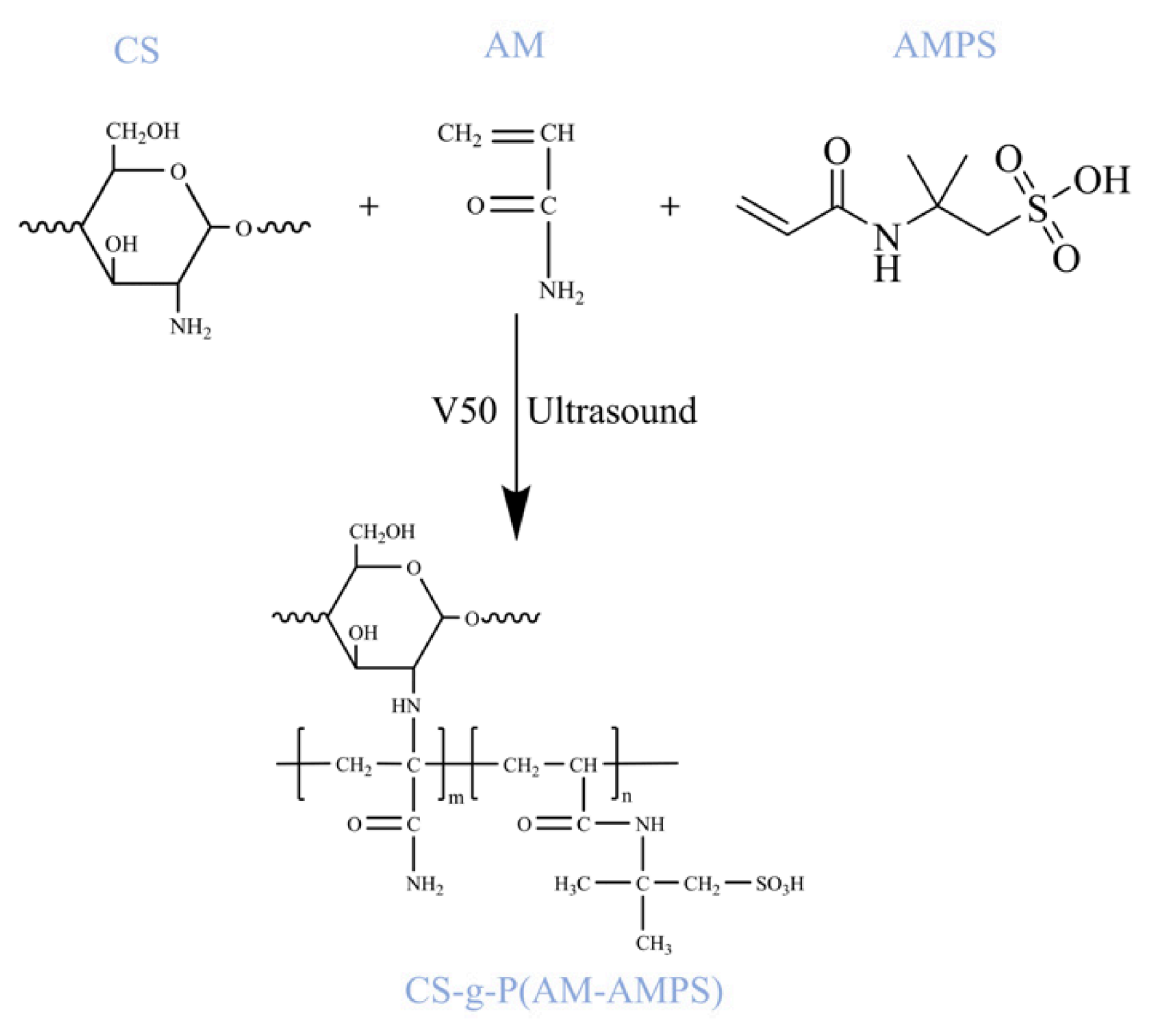
Schematic illustration of CS-g-P(AM-AMPS) synthesis pathway.

**Figure 3:**
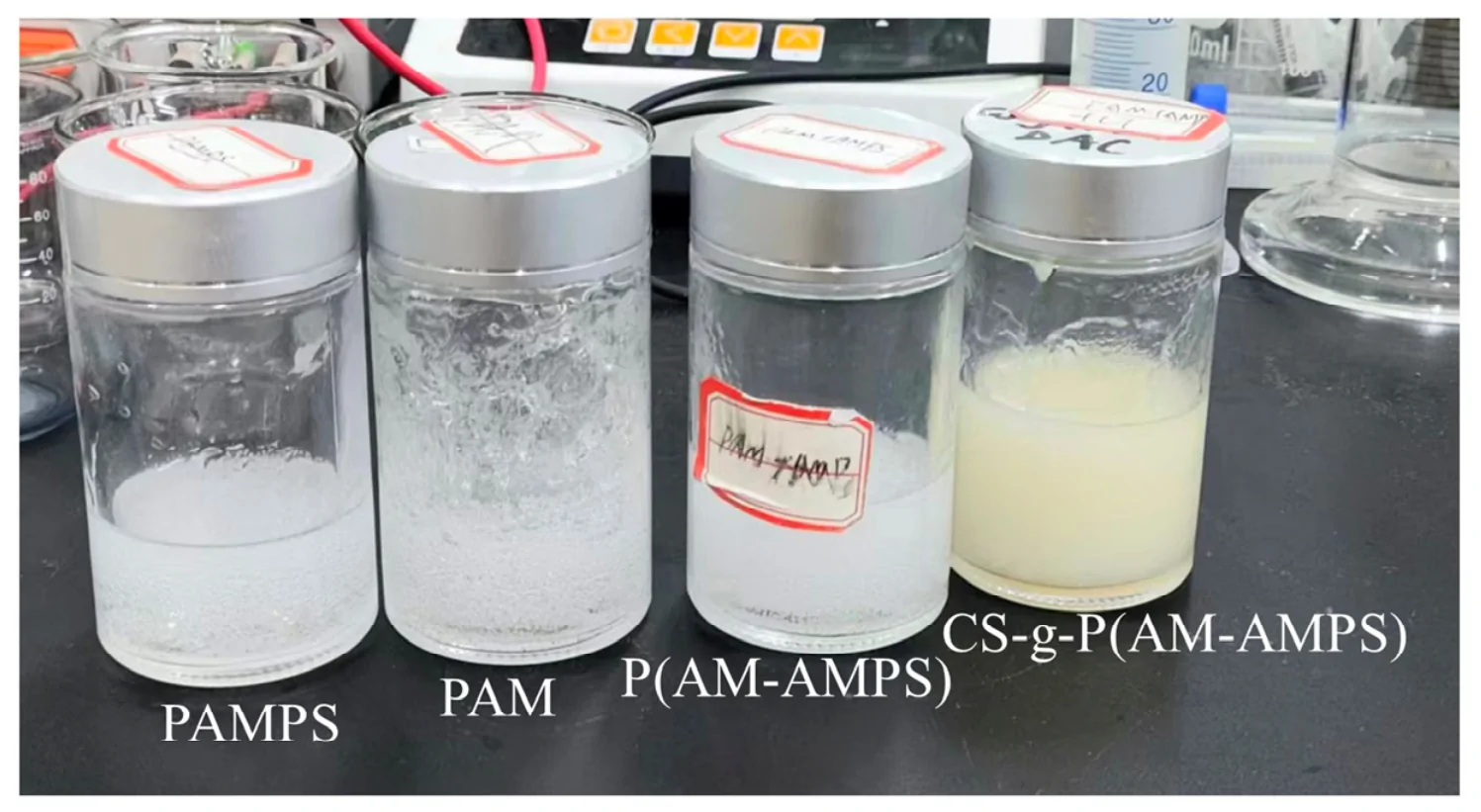
Comparison of polymer synthesis states.

The modified flocculant CS-g-P(AM-AMPS) was synthesized by graft copolymerizing AM and AMPS onto the CS backbone using 2,2′-Azobis(2-methylpropionamidine) dihydrochloride (V50) as the initiator in an ultrasonic water bath, as shown in Figure 1. First, a predetermined amount of chitosan was dissolved in a 1.0% dilute acetic acid solution to prepare a 5.0% chitosan stock solution for subsequent use. Then, a certain mass of AM and AMPS monomers, along with a specific volume of the 5.0% chitosan stock solution, was added into a 100 mL reaction flask and thoroughly shaken and stirred until completely dissolved. The molar feed ratio of AM, AMPS, and CS repeat units was approximately 7:1:1.44. Next, the pH of the reaction mixture was adjusted as required. To remove dissolved oxygen from the system and prevent its inhibitory effect on radical polymerization, high-purity nitrogen gas was continuously purged into the reaction flask for 20 min. After degassing, the predetermined amount of aqueous initiator V50 solution was quickly added, the flask was immediately sealed, and then placed into a constant-temperature ultrasonic water bath set at 90 °C for the graft copolymerization reaction. Upon completion of the designated reaction time, the ultrasonic device was turned off, the reaction flask removed, and allowed to stand and mature at room temperature for 2 h [20]. Subsequently, the product was repeatedly washed with anhydrous ethanol to reduce residual monomers and other soluble impurities. Finally, the purified product was dried in a forced-air oven set at 105 °C until constant weight was achieved, then ground into a powder and stored as modified chitosan flocculant. The synthetic pathways and states of the polymer products are illustrated in Figure 2 and Figure 3, respectively.

### 2.2. Experimental and Computational Methods

#### 2.2.1. Flocculation Test and Turbidity Analysis

To simulate actual water conditions and ensure suspension stability, 1.0 g of kaolinite powder was first added to 500 mL of deionized water to prepare a kaolinite suspension with a mass concentration of 2.0 g/L. The suspension was then subjected to 5 min of ultrasonic treatment to ensure complete dispersion of the kaolinite particles. Afterward, the suspension was left undisturbed for 24 h, and the upper layer was collected and diluted to the desired initial turbidity according to experimental requirements.

The prepared modified flocculant was dissolved in deionized water to form a stock solution at a concentration of 1.0 g/L, which was further diluted as needed for specific experiments. In batch flocculation tests, a predetermined volume of pre-treated kaolinite suspension was placed into a beaker, and the initial pH of the system was adjusted using either 0.1 mol/L HCl or NaOH solutions, focusing on the pH range of 2.0–7.0 to investigate its effect on the flocculant’s efficiency in removing kaolinite suspensions.

Flocculation tests were conducted using a TS6-1 programmable stirrer(Wuhan Hengling Technology Co., Ltd., Wuhan, China). In the dosage and pH experiments, polymer addition was followed by rapid mixing at 300 rpm for 2 min, slow mixing at 60 rpm for 60 min, and settling for 30 min. In the flocculation time experiment, the suspensions were mixed at 300 rpm for 2 min after polymer addition and then allowed to settle without further agitation for 5–30 min. Settling time was measured from the cessation of stirring. In each experiment, following the designated settling period, the supernatant was collected at a depth of approximately 2 cm below the liquid surface, and its residual turbidity was determined using a light-scattering turbidimeter(Hangzhou Qiwei Instrument Co., Ltd., Hangzhou, China).

The turbidity removal efficiency (*η*, %) was calculated using Equation (1):

(1) $$\eta \left(\%\right)=C_{0}-C_{e}/C_{0}\times 100\%$$

where *η* is the turbidity removal efficiency (%), and *C*_0_ and *C_e_* are the initial and residual turbidities of the kaolinite suspension, respectively, expressed in NTU.

#### 2.2.2. Physicochemical Characterization and Floc-Size Analysis

Fourier-transform infrared (FTIR) spectra of PAM, P(AM-AMPS), and CS-g-P(AM-AMPS) were recorded using a Nicolet iS20 spectrometer (Thermo Fisher Scientific, Waltham, MA, USA) over 500–4000 cm^−1^.

The weight loss of the samples was analyzed by a TG/DTA 8122 thermal analyzer from Rigaku, Tokyo, Japan, under a nitrogen atmosphere and a heating rate of 10 °C/min, over a temperature range of 30–600 °C.

After gold sputtering with an Emitech ion coater, the surface morphology of the samples was observed using a MIRA 3 LMS scanning electron microscope from TESCAN, Brno, Czech Republic. The SEM images of the polymer samples were analyzed and calculated using ImageJ software (version 1.53t, National Institutes of Health, Bethesda, MD, USA).

Particle size distribution of the samples was analyzed using a Mastersizer 2000 laser particle size analyzer(Malvern Instruments Ltd., Malvern, UK).

The turbidity of the samples was measured using a WGZ-1B turbidimeter produced by Hangzhou Qiwei Instrument Co., Ltd. (Hangzhou Qiwei Instrument Co., Ltd., Hangzhou, China).

The spatial complexity of the polymer surfaces was quantified from SEM images using the box-counting method implemented in ImageJ [28]. Images collected at the same magnification were converted to 8-bit binary images, and an identical thresholding procedure was applied to all samples. The number of boxes, *N*(*L*), required to cover the surface features was related to the box size, (*L*), by:

(2) $$N\left(L\right)=\;CL^{-D_{f}}$$

where *D_f_* is the box-counting fractal dimension and *C* is a proportionality constant. Logarithmic transformation gives:

ln(*N*) = −*D_f_* ln(*L*) + *C*_0_ (3)

Accordingly, *D_f_* was obtained from the absolute slope of the linear region in the ln *N*(*L*) versus ln*L* plot. The same box size intervals were used for each polymer throughout the process.

#### 2.2.3. Simulation Details

Molecular dynamics simulations were performed using the Forcite module in Materials Studio (version 2024, BIOVIA, Dassault Systèmes, San Diego, CA, USA) with the PCFF-INTERFACE force field. This force field has previously been used to model interactions between a polysaccharide-based graft flocculant and kaolinite [29]. The initial kaolinite structure was obtained from the American Mineralogist Crystal Structure Database and was geometrically optimized using the Castep module [30]. Because the kaolinite (001) plane is a commonly exposed basal surface, it was selected for constructing the interfacial model. The optimized unit cell was expanded into a 7 × 4 × 1 supercell with lateral dimensions of 36.07 × 35.77 Å^2^, and the resulting slab was oriented with its aluminol (Al-OH) basal face toward the hydrated polymer phase.

Three polymer models were constructed in Visualizer module. PAM was represented by a linear chain containing 80 AM repeat units. P(AM-AMPS) comprised 40 AM and 40 AMPS units arranged in a composition-constrained statistical sequence, providing a tractable atomistic representation of AM/AMPS copolymerization [31]. The CS-g-P(AM-AMPS) model consisted of a CS backbone containing 20 repeating units, consistent with the chain length previously adopted in atomistic simulations of chitosan [32], and four P(AM-AMPS) side chains, each comprising 10 AM and 10 AMPS units. The side chains were covalently attached to the C2 amino groups of CS units 3, 8, 13, and 18, numbered from one chain end. Selection of the C2 amino group was based on the grafting pathway identified in an analogous ultrasonically initiated chitosan graft-copolymerization system [33]. Because the grafting rate and the length and distribution of the side chains can influence the conformation and kaolinite adsorption of chitosan-based graft copolymers [34,35], the four grafting sites were distributed approximately uniformly along the CS backbone to reduce terminal effects and excessive local crowding. This construction fixed the number of vinyl-derived repeat units at 80 in all three models and maintained an AM/AMPS molar ratio of 1:1 in the two AMPS-containing polymers.

Each polymer was initially placed in an amorphous cell containing 1500 mobile water molecules. The hydrated polymer phase was positioned above the kaolinite (001) surface. An additional layer comprising 700 position-restrained water molecules was placed approximately 3 Å above the polymer solution layer to form a boundary buffer, and a 40 Å vacuum region was introduced normal to the surface to prevent interactions between periodic images.

Initial energy minimization was performed using the Smart algorithm at medium convergence for 20,000 steps. The minimized structures were annealed between 300 and 800 K for five cycles over a total duration of 250 ps to reduce dependence on the initial polymer configuration. The lowest-energy structure obtained from annealing was subsequently equilibrated in the NVT ensemble at 298 K using a Nosé–Hoover–Langevin thermostat. The integration time step was 1 fs.

**Figure 4:**
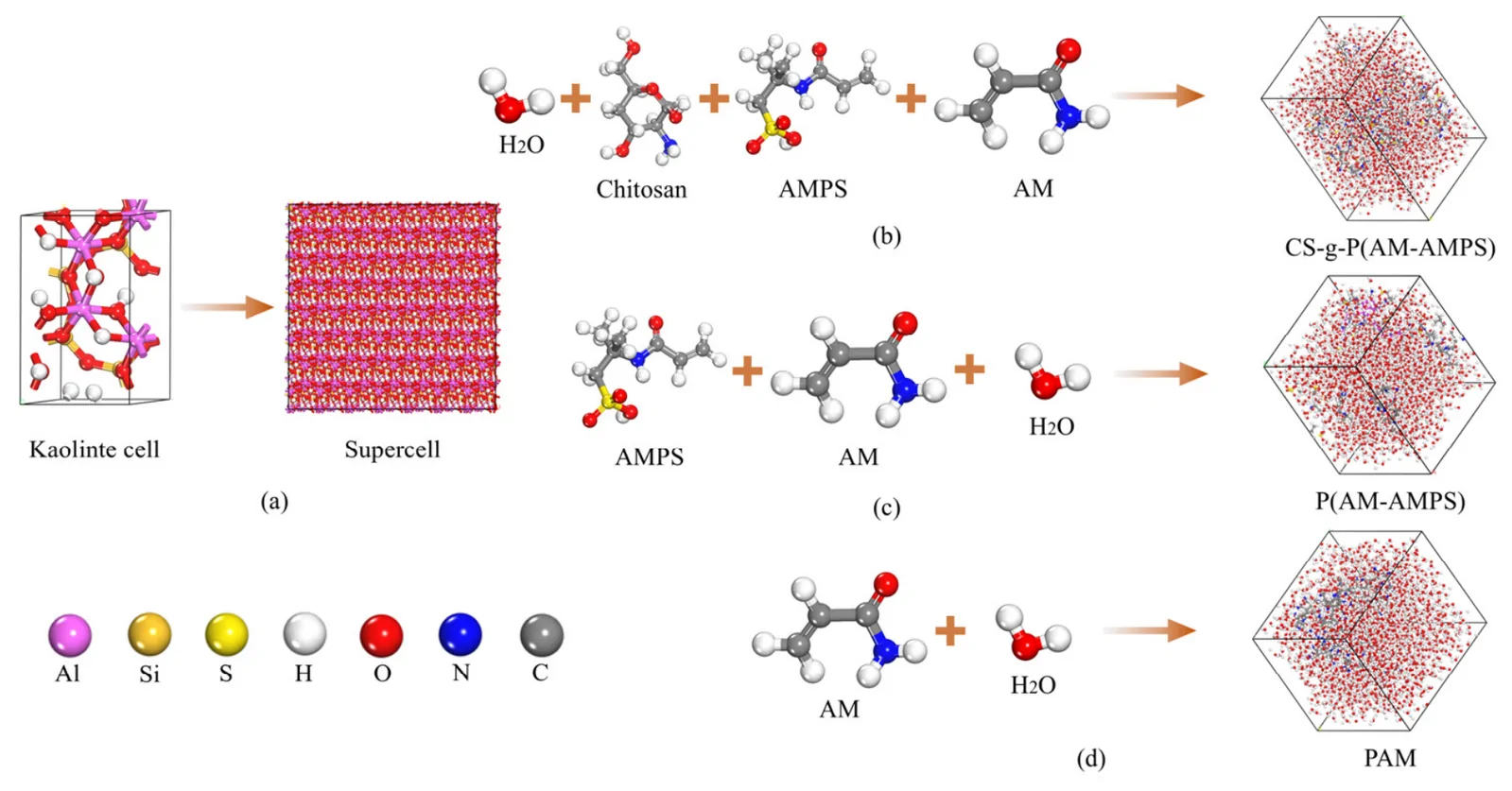
Molecular structure models: (**a**) kaolinite surface, (**b**) CS-g-P(AM-AMPS), (**c**) P(AM-AMPS), and (**d**) PAM.

**Figure 5:**
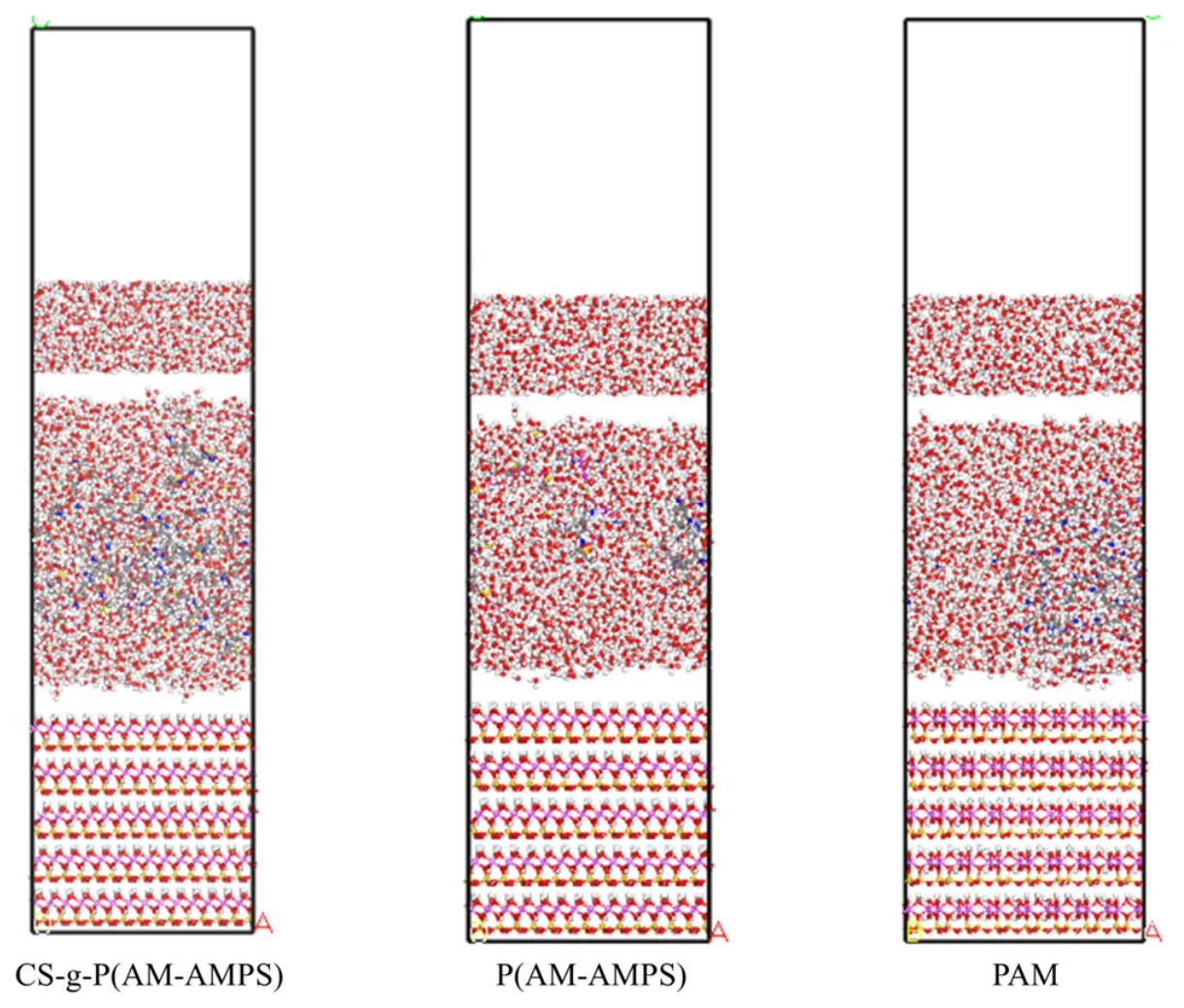
Molecular dynamics simulation model of molecules adsorbed at different polymer interfaces.

Electrostatic interactions were evaluated using the particle–particle particle–mesh (PPPM) method, whereas van der Waals interactions were calculated using an atom-based scheme with a cutoff distance of 12.5 Å. Each production simulation was run for 2000 ps and repeated three times, with the reported results averaged over the three trajectories. Figure 4 and Figure 5 show the molecular structures and interfacial models, respectively.

#### 2.2.4. Calculation of Molecular Simulation Descriptors

The extent of the interactions of polymers units on the kaolinite surfaces can be demonstrated by the interaction energy. The interaction energy was calculated as follows:

*E_interaction_* = *E_total_* − *E_polymer_* − *E_kao_*_(001)_ (4)

where *E_total_* is the energy of the complete polymer–kaolinite system, and *E_polymer_* and *E_kao_*_(001)_ represent the energies of the isolated polymer and the kaolinite surface, respectively, when the adsorbed configuration is separated. A more negative *E_interaction_* indicates a more energetically favorable polymer-surface association within the adopted model [23].

The radius of gyration (*R_g_*) is used to describe the molecular size of polymers in aqueous solution and the extent of extension of flexible segments [36]. Its physical meaning is the root-mean-square distance of all atoms within a molecule from their common center of mass, calculated by the following formula:

(5) $$R_{g}=\sqrt{\frac{\sum_{i=1}^{N}m_{i}s_{i}^{2}}{\sum_{i=1}^{N}m_{i}}}$$

in the equation, *m_i_* represents the mass of the *i*-the atom in the copolymer, *s_i_* denotes the distance from that atom to the center of mass of the entire polymer, and *N* is the total number of atoms in the polymer. A larger *R_g_* value indicates a more extended conformation of the polymer molecule in solution, which favors the formation of larger flocs.

Polymer diffusion was evaluated using the mean square displacement (MSD) and diffusion coefficient (*D*) [37,38]. The MSD was calculated as follows:

(6) $$\mathrm{MSD}=\frac{1}{N}\sum_{i=1}^{N}{\;\left[r_{i}\left(t\right)-r_{i}\left(0\right)\right]}^{2}$$

where *r_i_*(*t*) and *r_i_*(0) denote the position vectors of atom *i* at time *t* and at the initial time, respectively, and *N* is the number of polymer atoms included in the analysis.

Based on the MSD versus time curve after the system reaches equilibrium, the diffusion coefficient of the molecules in solution can be calculated by determining the slope *K_MSD_* during the linear fitting stage:

(7) $$D=\frac{1}{6}K_{\mathrm{MSD}}$$

A higher apparent diffusion coefficient may facilitate initial contact between polymer chains and suspended particles, but the final flocculation efficiency also depends on interfacial anchoring strength and chain extension [39].

The spatial distributions of polymer functional groups and water molecules along the z direction, normal to the kaolinite surface, were characterized using relative concentration profiles [40]. For species *j*, the local number density and relative concentration were calculated as follows:

(8) $$\rho_{j}\left(z\right)=\frac{N_{j}\left(z\right)}{A\Delta z},\;C_{\mathrm{rel},j}\left(z\right)=\frac{\rho_{j}\left(z\right)}{{\bar{\rho }}_{j}}$$

where *N_j_*(*z*) is the number of selected atoms or molecules within a slab of thickness Δz. *A* is the cross-sectional area of the simulation cell perpendicular to the z direction, and ${\bar{\rho }}_{j}$ is the mean number density of species *j* in the simulation system.

The number of hydrogen bonds between each polymer and the kaolinite (001) surface was calculated from the equilibrated trajectories. A hydrogen bond was identified when the distance between the donor-bound hydrogen atom and the acceptor atom was less than 3.0 Å and the donor-hydrogen-acceptor (*D*-*H*···*A*) angle exceeded 120° [41]. Hydrogen bonds with either the polymer or the kaolinite surface acting as the donor were included.

## 3. Results and Discussion

### 3.1. Physicochemical Characterization of the Polymer Products

#### 3.1.1. FTIR Analysis

**Figure 6:**
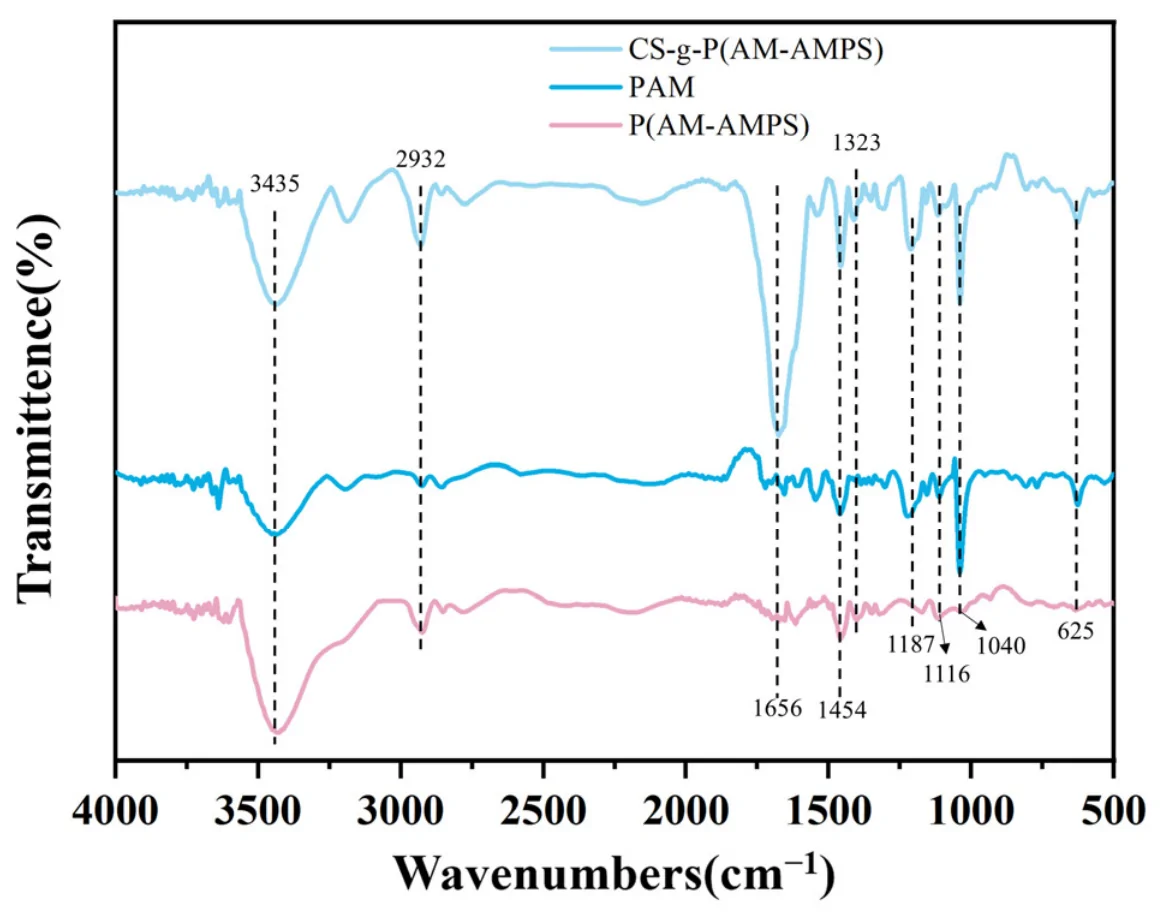
FTIR spectra of CS-g-P(AM-AMPS), P(AM-AMPS), and PAM.

Figure 6 compares the FTIR spectra of CS-g-P(AM-AMPS), P(AM-AMPS), and PAM. In the spectrum of CS-g-P(AM-AMPS), the broad band at 3435 cm^−1^ is attributed to overlapping O-H and N-H stretching vibrations. This absorption lies in the O-H/N-H region reported for pure chitosan, with bands near 3416 cm^−1^ [42] and 3440 cm^−1^ in the CTS-DAC-AMPS flocculant study by Liu et al. [43]. The band at 2932 cm^−1^ corresponds to asymmetric CH_2_ stretching, while the band at 1454 cm^−1^ is assigned to CH_2_ bending. The band at 1656 cm^−1^ is consistent with amide C=O stretching [17,44]. Contributions may arise from AM-and AMPS-derived amide groups and residual N-acetyl groups in CS [42].

The absorption near 1040 cm^−1^ may partly arise from AMPS-derived sulfonate groups [45], similar to the band reported at 1038 cm^−1^ for CTS-DAC-AMPS [43]. C-O and C-O-C vibrations of the CS backbone may also overlap in this region [42]. The band at 625 cm^−1^ is tentatively assigned to C-S stretching, as reported by Xu et al. [46] for an AMPS-containing polymer/nano-silica composite. The O-H/N-H and amide-related bands, along with these fingerprint-region absorptions, are consistent with the expected functional-group composition of CS-g-P(AM-AMPS). The XPS results in Section 3.1.3 also support the presence of AMPS-derived sulfonate groups.

#### 3.1.2. Thermal Analysis

**Figure 7:**
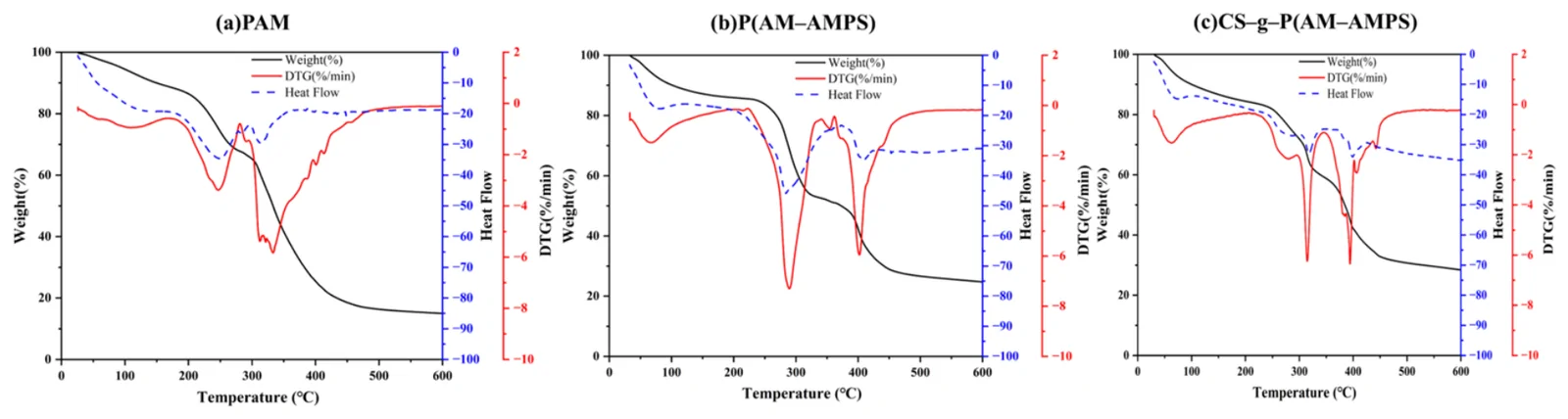
TGA-DYG-DSC curves.

The thermal stability of the polymers was investigated using thermogravimetric analysis (TGA), derivative thermogravimetry (DTG), and differential scanning calorimetry (DSC), with the results shown in Figure 7. From the data, all three synthesized polymers exhibit three distinct weight-loss stages.

The first stage of weight loss is attributed to the evaporation of free water and bound water within the polymer matrix [33,47]. According to the DTG curves, the end temperatures of the first-stage weight loss for CS-g-P(AM-AMPS), P(AM-AMPS), and PAM were approximately 150, 200, and 200 °C, with corresponding weight losses of approximately 18%, 15%, and 15%, respectively.

The second stage involves not only partial decomposition of the CS backbone but also thermal degradation and imidization reactions of the amide groups in AM and AMPS molecules [42,48]. The peak temperatures of maximum weight loss rate in this stage can be determined from the minima on the DTG curve: for CS-g-P(AM-AMPS), the peak temperature is about 315 °C, accompanied by a distinct endothermic valley in the heat flow curve, with a weight loss of approximately 22%. For P(AM-AMPS), the peak temperature is around 290 °C, with a weight loss of about 30%. In contrast, PAM exhibits the lowest peak temperature at approximately 240 °C, with a weight loss of about 25%.

The third stage of weight loss is primarily due to the breakdown of C-C and C-N bonds in the main chains of CS, AM, and AMPS within the polymer products. For CS-g-P(AM-AMPS), the characteristic DTG peak of this stage appears at around 395 °C, with a weight loss of approximately 32%, and the highest residual mass of about 28% remains at 600 °C. For P(AM-AMPS), the weight loss during this stage is about 30%, leaving a final residue of approximately 25%. Meanwhile, PAM undergoes continuous and intense decomposition near 330 °C and 400 °C, resulting in a significant weight loss of up to 45% and a final residue of only about 15%.

Overall, CS-g-P(AM-AMPS) exhibited three weight-loss stages similar to those of P(AM-AMPS) and PAM. Its higher main decomposition peak temperature (approximately 315 °C) and greater residual mass indicate improved thermal stability compared with the reference polymers. Regarding storage performance, Shi et al. [49] reported that a g-C_3_N_4_-cross-linked chitosan/PAM flocculant retained its flocculation efficiency after 90 days under dark and dry conditions, with negligible deterioration. This provides a relevant experimental precedent for investigating the storage potential of CS-g-P(AM-AMPS).

#### 3.1.3. XPS Analysis

**Figure 8:**
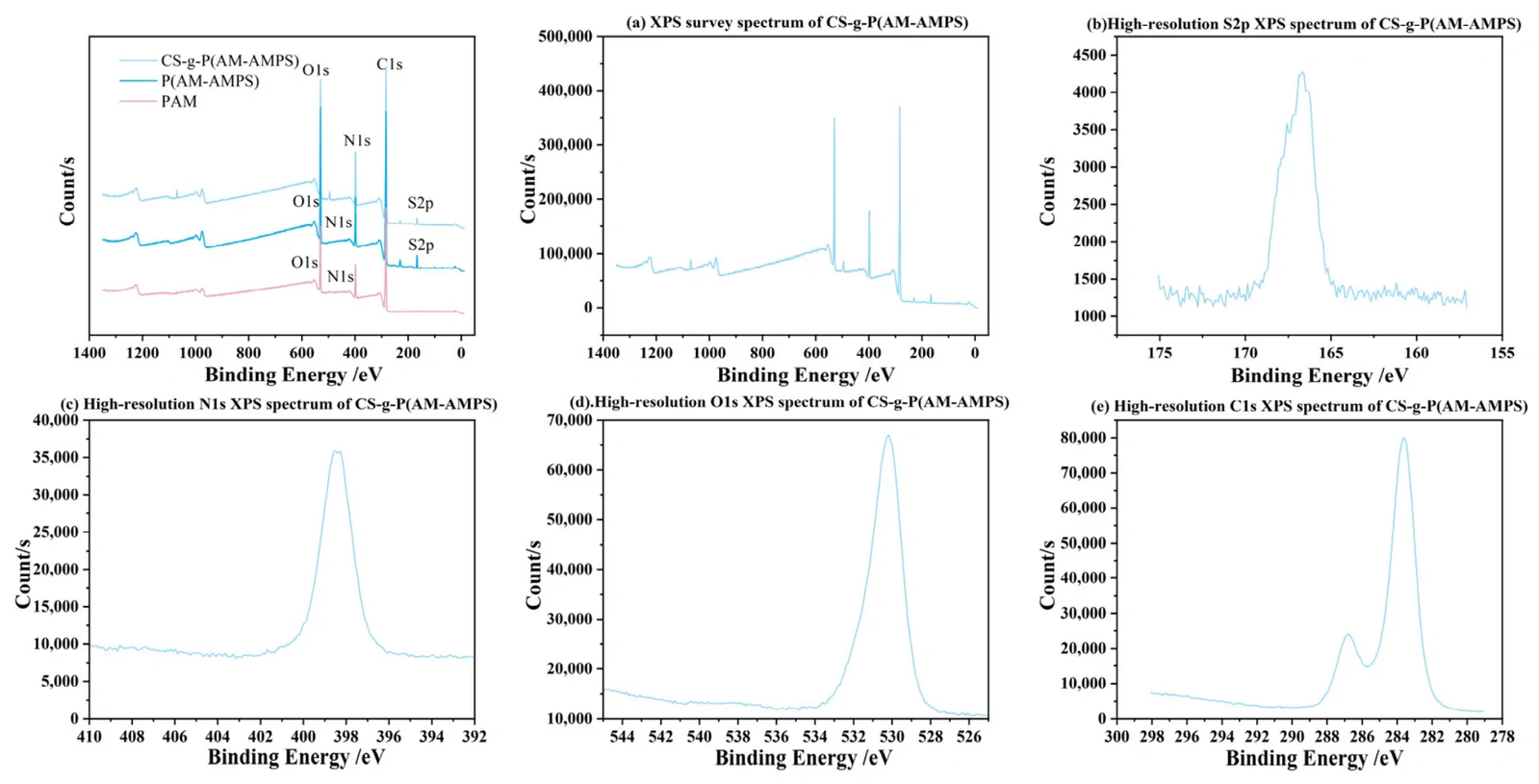
XPS spectra: survey comparison of the three polymers (upper left); (**a**) survey and (**b**–**e**) high-resolution S 2p, N 1s, O 1s, and C 1s spectra of CS-g-P(AM-AMPS), respectively.

XPS characterization of the graft copolymer CS-g-P(AM-AMPS), P(AM-AMPS), and PAM was performed, focusing primarily on the elements C, N, O, and S, with results shown in Figure 8.

Figure 8 and Table 1 compare the surface elemental compositions of CS-g-P(AM-AMPS), P(AM-AMPS), and PAM. PAM shows C, N, and O signals, with no detectable sulfur, whereas both AMPS-containing polymers exhibit an S 2p signal near 168.0 eV. The surface sulfur contents of CS-g-P(AM-AMPS) and P(AM-AMPS) are 1.29% and 2.29%, respectively. Given the sulfur-free structures of CS and PAM, the sulfur detected in CS-g-P(AM-AMPS) supports the presence of AMPS-derived groups in the chitosan-based product. This elemental evidence complements the FTIR assignment of AMPS-related sulfonate absorption, particularly in the fingerprint region where sulfur-oxygen vibrations overlap with CS-related C-O and C-O-C vibrations.

High-resolution XPS spectra were further fitted to examine the surface chemical environments of CS-g-P(AM-AMPS) (Figure 8b–e). The S 2p signal at 167.8 eV is consistent with sulfonate sulfur in the AMPS-containing segments. In their study of poly(OxMA-co-SBMA)-modified surfaces, Chou and Ou observed a sulfonate-related S 2p signal after grafting, whereas no sulfur signal was detected on the APTES-modified control. They also reported an N^+^:SO_3_^−^ ratio of 1.0906 for the grafted surface, close to the 1:1 stoichiometry of the sulfobetaine groups [50]. This comparison supports using the sulfur signal as evidence for the introduction of sulfonate-containing segments. The N 1s signal at 399.4 eV is consistent with amide nitrogen, with possible contributions from residual amino groups of CS, while the O 1s envelope near 531.2 eV reflects overlapping oxygen-containing environments, including carbonyl and sulfonate groups. The C 1s spectrum was resolved into components at 284.6 eV (C-C/C-H), 286.3 eV (C-O), and 287.8 eV (amide C=O). For an AM/AMPS-grafted starch flocculant, Kang et al. reported S 2p and N 1s signals near 169 and 400 eV, respectively, together with an amide N-C=O component at 288.0 eV [51]. The proximity of this amide component to the 287.8 eV component observed here supports the amide carbonyl assignment. Together, the XPS and FTIR results support the expected functional-group composition of CS-g-P(AM-AMPS).

#### 3.1.4. SEM Analysis

**Figure 9:**
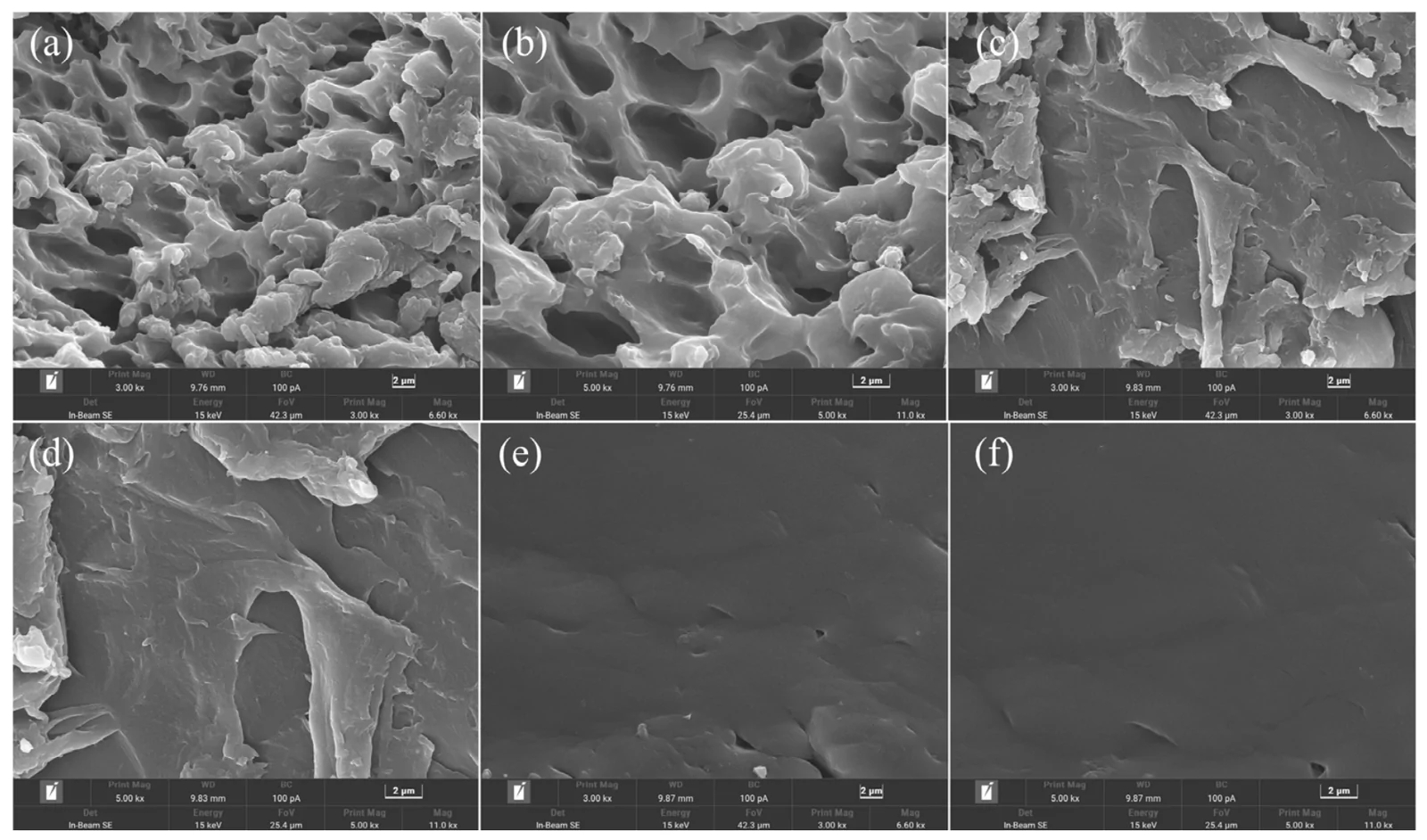
SEM analysis results of CS-g-P(AM-AMPS), P(AM-AMPS), and PAM; (**a**,**b**) images of CS-g-P(AM-AMPS) at 3000× and 5000× magnification; (**c**,**d**) images of P(AM-AMPS) at 3000× and 5000× magnification; (**e**,**f**) images of PAM at 3000× and 5000× magnification.

**Figure 10:**
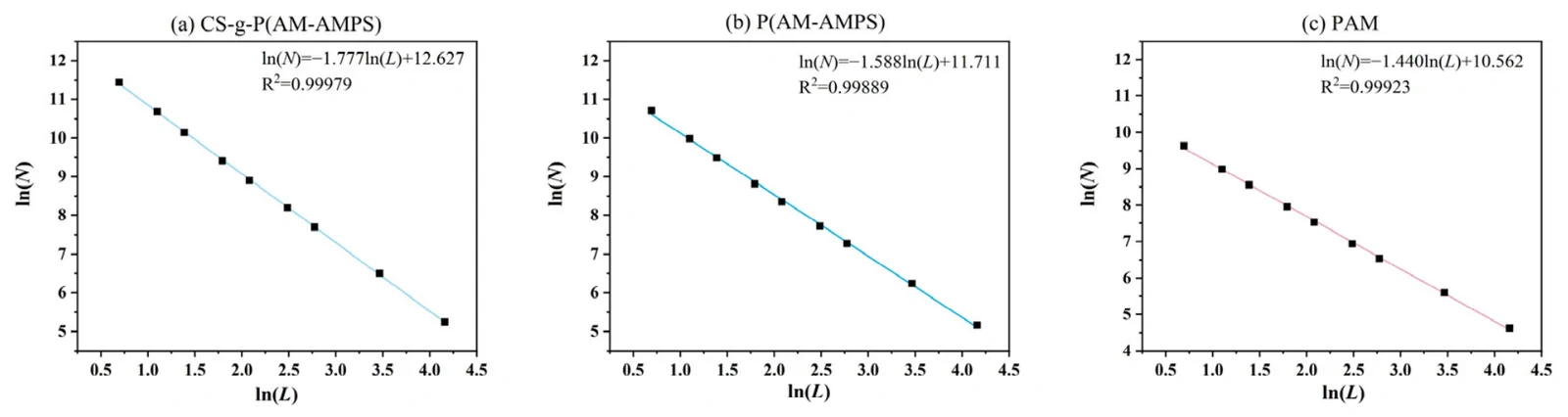
Fractal dimensions of CS-g-P(AM-AMPS), P(AM-AMPS), and PAM.

The SEM images and fractal dimension analysis results of the graft copolymer CS-g-P(AM-AMPS), as well as P(AM-AMPS) and PAM, are shown in Figure 9 and Figure 10.

From Figure 9, it can be observed that the surface morphologies of these three polymers exhibit certain differences. The surface of PAM is very smooth, with a relatively dense and flat structure; the surface of the copolymer P(AM-AMPS) shows slight wrinkles and protrusions, making it overall rougher; the grafted copolymer CS-g-P(AM-AMPS) has the roughest surface, featuring numerous pores and grooves, and appears to have a more developed rough and porous surface among the three copolymers.

The rougher morphology of CS-g-P(AM-AMPS) is plausibly associated with disruption of the ordered chitosan packing and the introduction of compositionally distinct grafted segments. A comparable architecture-dependent effect has been reported for amphiphilic chitosan-g-polyisobutylene copolymers, in which increasing graft number and branch length weakened chitosan-backbone crystallization and increased surface roughness [52]. Although the graft chemistry differs from the present system, that result supports interpreting the observed wrinkles and pores as a structural consequence of grafting. Fractal analysis was therefore used here to quantify the two-dimensional morphological complexity of the SEM images [53].

The linear fits in Figure 10 gave correlation coefficients above 0.998, indicating strong scale-dependent self-similarity over the analyzed image range. CS-g-P(AM-AMPS) had the highest fractal dimension (*D_f_* = 1.777), followed by P(AM-AMPS) (1.588) and PAM (1.440), consistent with the qualitative SEM ranking of surface irregularity. The larger *D_f_* demonstrates greater two-dimensional morphological complexity in the analyzed dry-state images. It does not, by itself, quantify the hydrated specific surface area, the number of accessible adsorption sites, or adsorption capacity. The proposed link between rough morphology and flocculation should therefore be considered jointly with the interfacial-energy, chain-conformation, and performance results rather than treated as an independent proof of stronger adsorption.

### 3.2. Flocculation Performance

#### 3.2.1. Effect of Polymer Dosage

The dosage of flocculant is one of the key factors determining flocculation efficiency [2,38]. When the dosage is too low, the total number of active sites available for electrostatic attraction and adsorption bridging in the system is insufficient to effectively capture all suspended particles, resulting in low coagulation efficiency. Conversely, excessive dosage can lead to entanglement of polymer chains in solution, creating strong steric hindrance that hinders full extension of the molecular chains [54]. Additionally, according to the charge neutralization theory, an excess of cationic flocculant macromolecules adsorbed onto negatively charged kaolinite particle surfaces may cause charge reversal. In this case, the encapsulated particles acquire like positive charges, leading to renewed strong electrostatic repulsion between particles. This results in re-stabilization of previously destabilized suspended particles, which macroscopically manifests as a decrease in turbidity removal efficiency [1].

**Figure 11:**
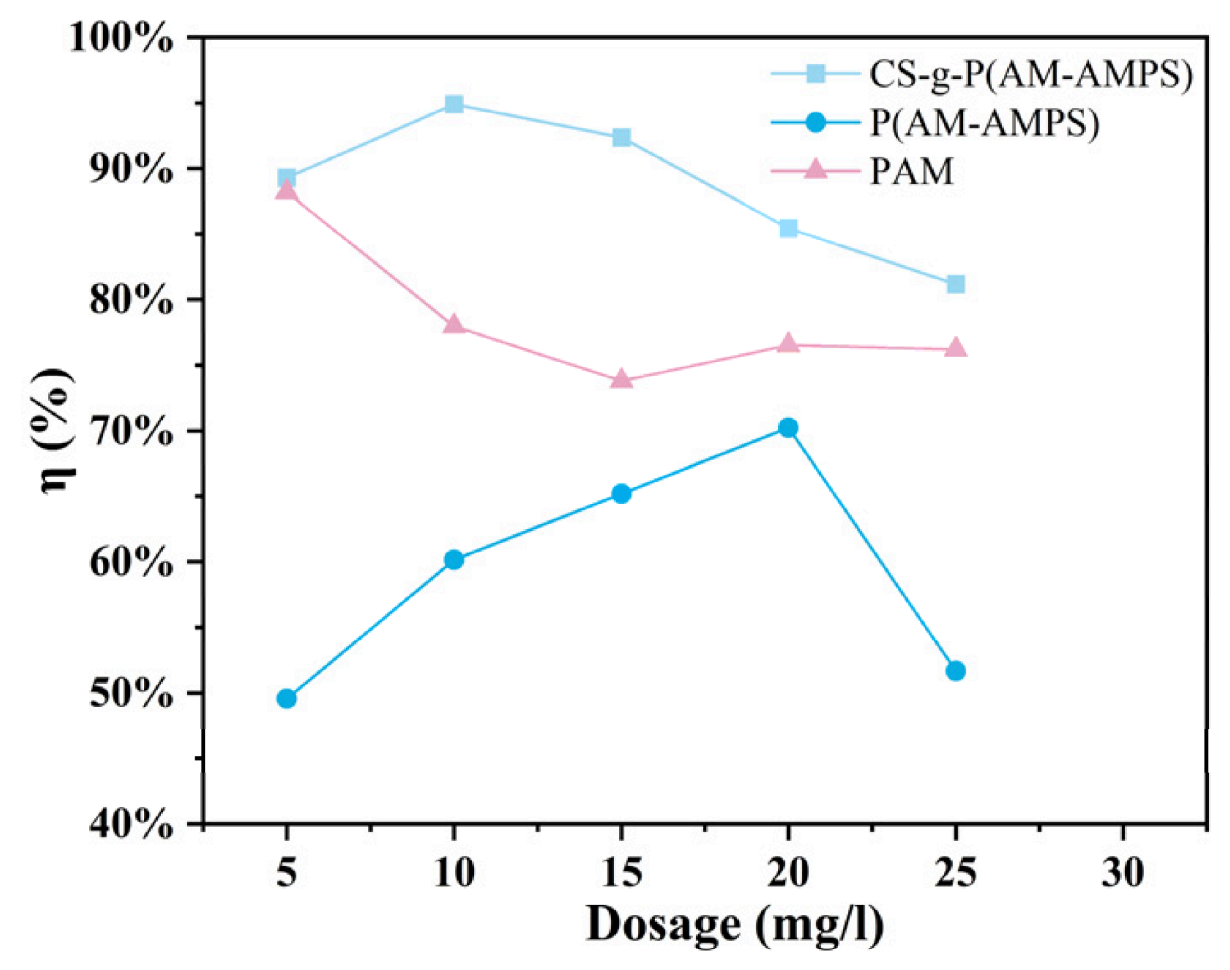
Influence of CS-g-P(AM-AMPS), P(AM-AMPS), and PAM dosage on turbidity removal at an initial pH of 6.0.

To simulate the natural weakly acidic to neutral environment of typical slope groundwater, the initial pH of the system was uniformly adjusted to 6.0. Under these conditions, a systematic investigation was conducted into the effects of CS-g-P(AM-AMPS), P(AM-AMPS), and PAM dosages on the removal efficiency of kaolinite suspensions (results are presented in Figure 11). Experimental results indicated that as the flocculant dosage increased, the removal efficiency of suspended solids initially rose rapidly and then gradually declined, exhibiting a distinct optimal dosage range effect. The optimal dosages for CS-g-P(AM-AMPS), P(AM-AMPS), and PAM were found to be 10.0 mg/L, 20.0 mg/L, and 5.0 mg/L, respectively, corresponding to maximum removal rates of 94.9%, 70.2%, and 88.2%. Although all three materials demonstrated certain flocculation capabilities under model kaolinite suspensions at pH 6.0, the newly developed graft copolymer CS-g-P(AM-AMPS) showed significantly superior turbidity removal performance compared to the other two. This result suggests that the amphoteric structure and extended molecular configuration of CS-g-P(AM-AMPS) contribute to its enhanced flocculation performance.

#### 3.2.2. Effect of Solution pH

**Figure 12:**
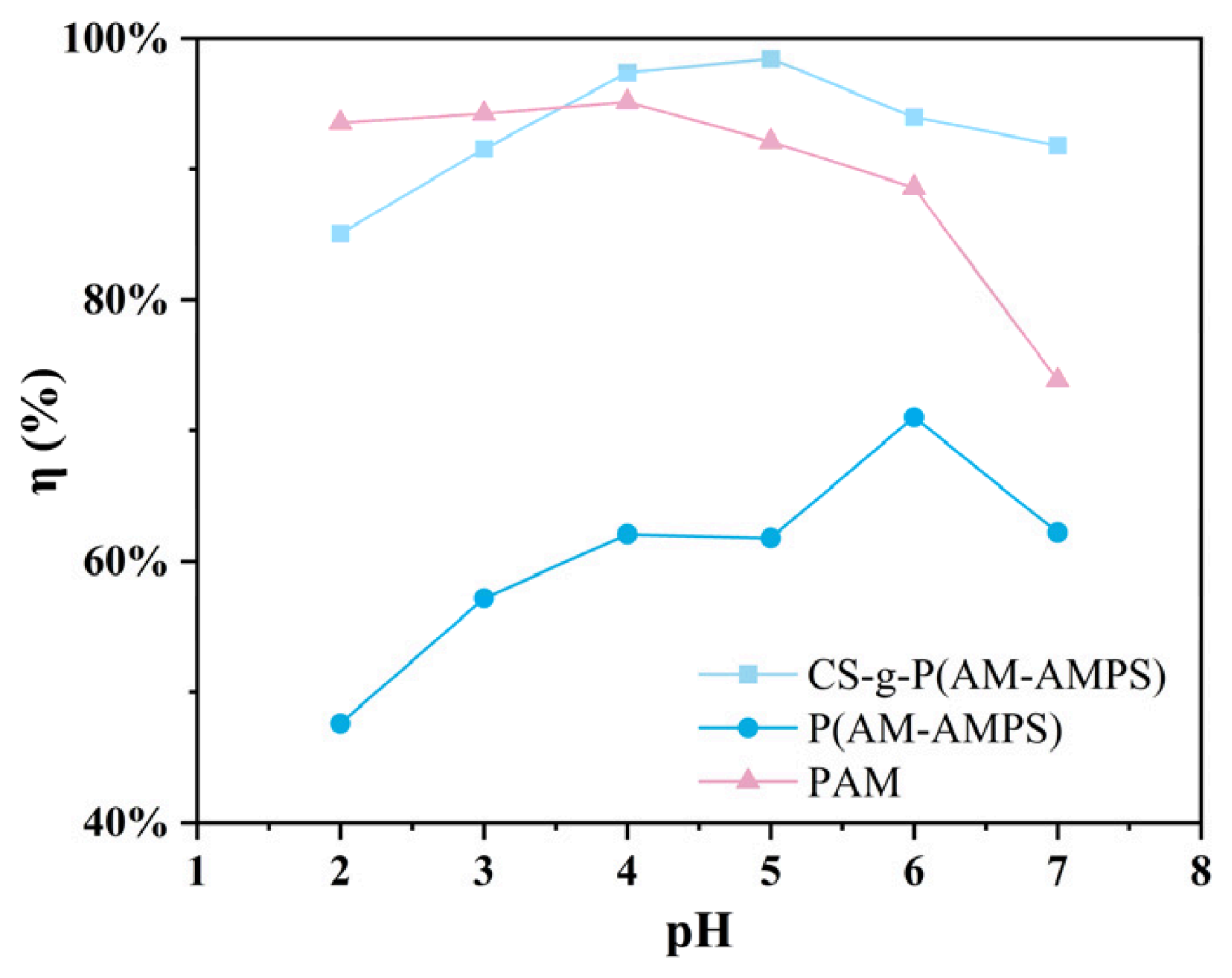
Influence of pH.

The effect of pH on the efficiency of flocculant in removing kaolinite suspension was studied using the optimal dosage for each group of flocculants, and the results are shown in Figure 12.

CS-g-P(AM-AMPS) demonstrated excellent removal efficiency across a pH range of 2.0–7.0, achieving the highest removal rate (η = 98.5%) at pH 5.0–3.4 percentage points higher than PAM’s maximum removal rate (η = 95.1%) at pH 4.0. This indicates that the synthesized copolymer is highly effective in removing suspended particles.

Under strongly acidic conditions (pH 2~3), CS-g-P(AM-AMPS) showed lower removal efficiency compared to PAM; however, its performance was superior at pH 4.0~7.0. The flocculation and sedimentation ability of the grafted copolymer is less sensitive to pH changes, although it performs relatively better under acidic conditions. This behavior can be explained by the combined contributions of amino-group protonation, hydrogen bonding, and graft-chain conformation: first, under acidic conditions, the amino groups on the chitosan backbone can be protonated, forming positively charged ammonium groups, which can contribute to charge neutralization at negatively charged kaolinite sites and promote polymer-particle association, consistent with findings for related chitosan-acrylamide flocculants [55,56]; second, the amide groups in the grafted AM segments can participate in hydrogen bonding with surface hydroxyl groups and interfacial water molecules, while the sulfonate groups in AMPS promote hydration and electrostatic extension of the graft chains.

The relatively high removal efficiency of PAM at some pH values may reflect the accessibility and flexibility of its linear chains, which facilitate contact with kaolinite particles and interparticle bridging. In contrast, P(AM-AMPS) showed the lowest overall removal efficiency. This behavior may be associated with the strongly hydrated, anionic sulfonate groups pendant to the copolymer backbone, which can restrict close approach to negatively charged sites on kaolinite through electrostatic repulsion and persistent hydration [57].

#### 3.2.3. Effect of Settling Time

**Figure 13:**
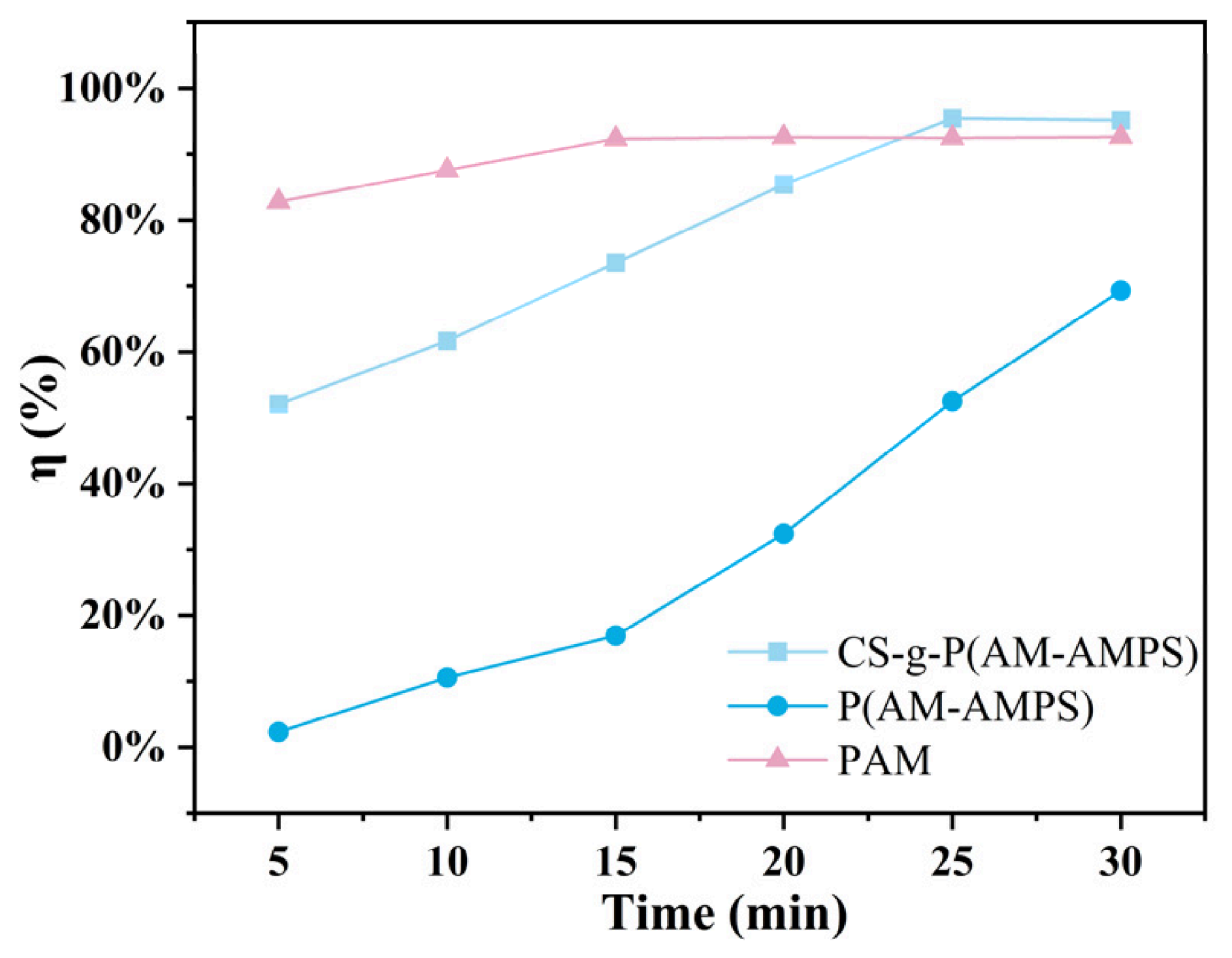
Influence of settling time.

Figure 13 shows the effect of settling time (5–30 min) on turbidity removal from kaolinite suspensions at the respective optimal polymer dosages and pH values. Removal efficiency increased with settling time, but the three polymers exhibited different response profiles. PAM and CS-g-P(AM-AMPS) approached plateaus within the tested period, whereas P(AM-AMPS) continued to increase up to 30 min.

PAM achieved 82.7% removal after 5 min and showed limited further improvement after approximately 15 min. By contrast, CS-g-P(AM-AMPS) exhibited lower removal efficiency at early settling times but continued to improve over a longer period, reaching 95.4% at 25 min with little further change at 30 min. Thus, the grafted polymer required a longer settling period to attain its higher final removal efficiency.

P(AM-AMPS) exhibited the lowest removal efficiency throughout the tested settling period, reaching 69.2% at 30 min. Its continued increase indicates that turbidity removal had not reached a plateau within the investigated time range. For CS-g-P(AM-AMPS), considering both removal efficiency and treatment duration, 25 min was selected as the optimal time for turbidity removal.

#### 3.2.4. Effect of Polymer Type on Floc Size Distribution

**Figure 14:**
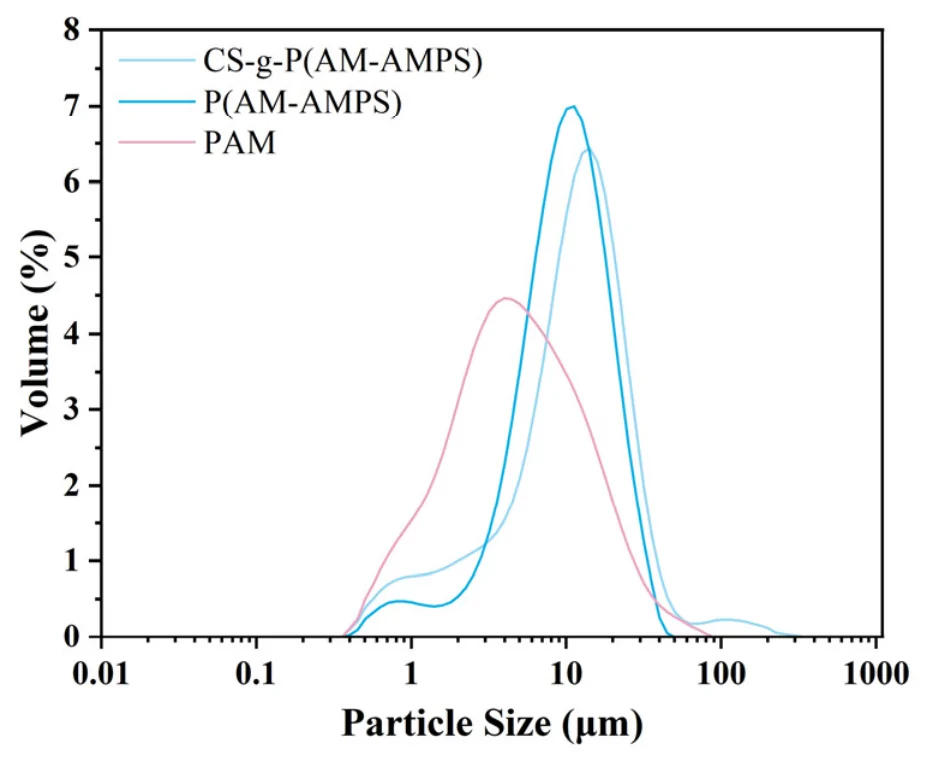
Particle size distribution of kaolinite flocs under optimal conditions.

Floc particle size is an important structural parameter affecting solid–liquid separation and sedimentation performance [58]. According to Stokes’ sedimentation law, an increase in particle diameter generally results in a higher terminal settling velocity when the particle-fluid density difference and fluid properties are comparable [59]. Figure 14 compares the floc particle-size distributions obtained under the optimal conditions for the three polymers. The median floc sizes (*d*_50_) after treatment with CS-g-P(AM-AMPS), P(AM-AMPS), and PAM were 10.89, 9.26, and 4.48 μm, respectively.

CS-g-P(AM-AMPS) produced the largest flocs, with a *d*_50_ approximately 2.5 times that obtained with linear PAM. Its particle-size distribution was also shifted toward larger sizes relative to that of PAM, indicating more extensive aggregation of fine kaolinite particles under the tested conditions. This increase in floc size may be associated with the grafted polymer architecture, which provides multiple adsorption sites and an extended bridging range for particle capture and floc growth [16]. By comparison, the smaller *d*_50_ obtained with PAM indicates the formation of comparatively smaller aggregates. These results show that CS-g-P(AM-AMPS) promoted the formation of larger kaolinite aggregates under its selected test conditions. P(AM-AMPS) produced a larger median floc size than PAM but achieved lower turbidity removal, showing that median floc size alone does not determine clarification performance.

### 3.3. MD Analysis

#### 3.3.1. Interface Interaction Energy Analysis

**Figure 15:**
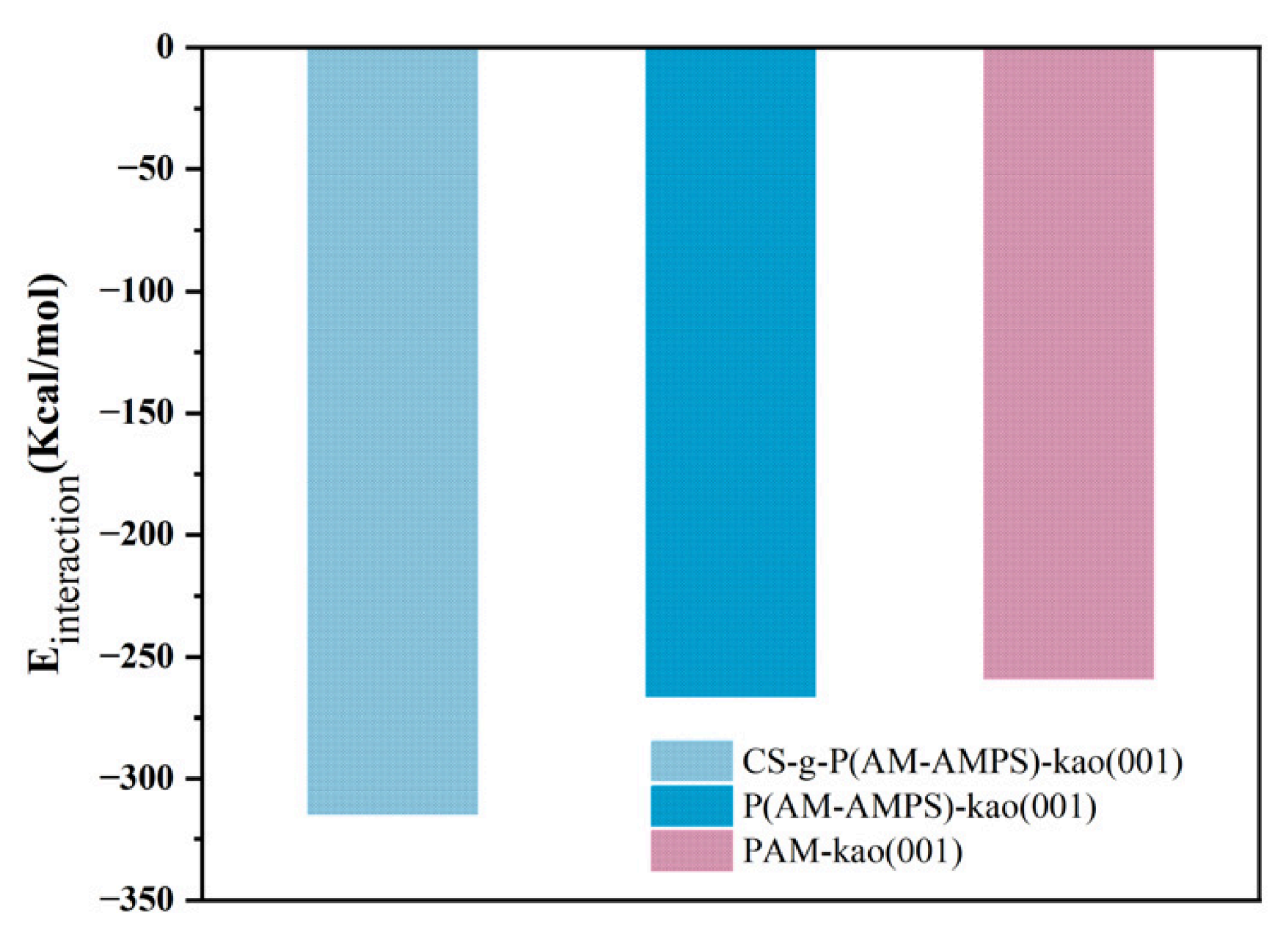
Interaction energies between CS-g-P(AM-AMPS), P(AM-AMPS), and PAM with the kaolinite (001) surface.

As illustrated in Figure 15 and Table 2, CS-g-P(AM-AMPS) had the most negative interaction energy with the kaolinite (001) surface (−314.674 kcal/mol), followed by P(AM-AMPS) (−266.396 kcal/mol) and PAM (−258.935 kcal/mol). These values describe the total interaction of each polymer model with the kaolinite surface. The more negative energy of CS-g-P(AM–AMPS) reflects contributions from its molecular size, functional-group composition and the extent of surface contact. The enhanced affinity is consistent with the coexistence of amino groups, hydroxyl groups, amide groups, and sulfonate groups, which provide multiple opportunities for electrostatic attraction, hydrogen bonding, and hydrated interfacial association. Interaction energy is a comparative energetic descriptor and should not be interpreted alone as a measure of adsorption rate or bridging force. It represents a potential-energy difference rather than an adsorption free energy. The polymers were simulated in their initially constructed chemical forms with fixed protonation states. The energy comparison therefore does not capture changes in interfacial interactions arising from pH-dependent protonation. Direct chi-g-PAM/kaolinite experiments have shown that adsorption and bridging also depend on surface chemistry, ionic environment, and chain conformation [24]. The following analyses therefore examine whether the energy ranking is accompanied by favorable extension and spatial distribution.

#### 3.3.2. Radius of Gyration and Mean Square Displacement Analysis

**Figure 16:**
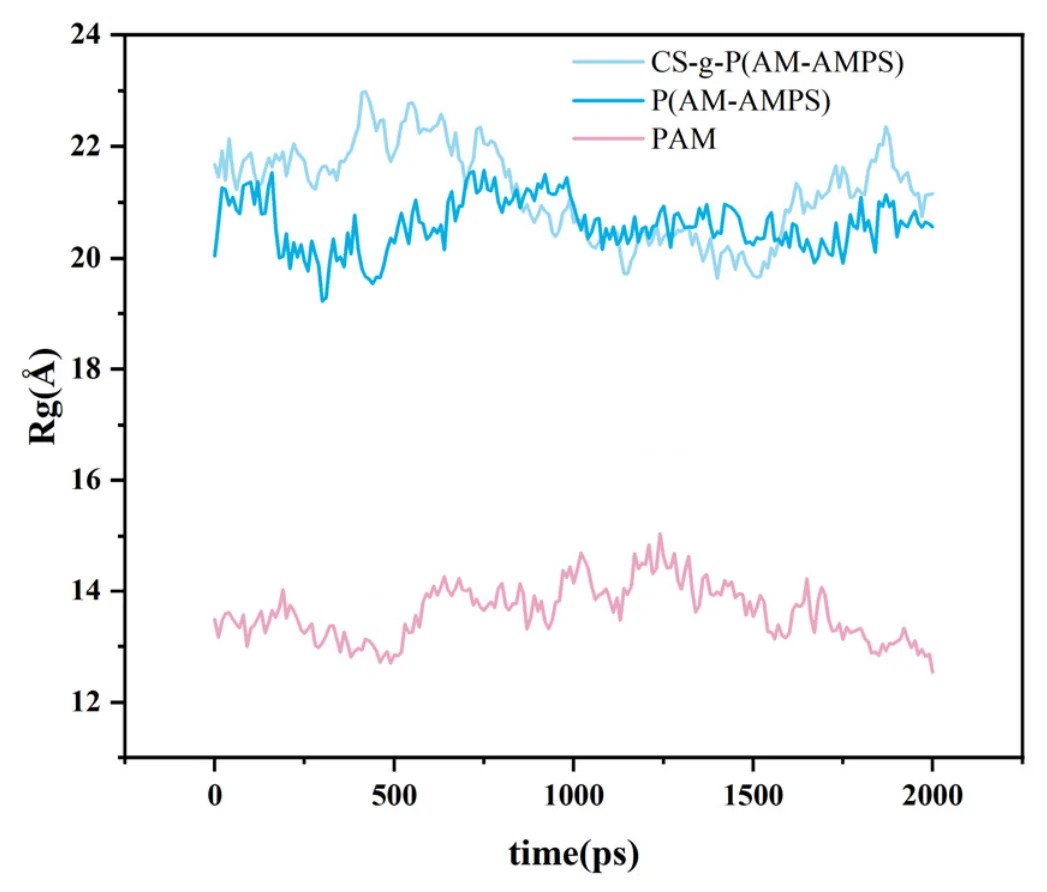
Radius of gyration of CS-g-P(AM-AMPS), P(AM-AMPS), and PAM.

As shown in Figure 16 and Table 3, statistical calculations performed on the dynamic equilibrium trajectories yield average radii of gyration for CS-g-P(AM-AMPS), P(AM-AMPS), and PAM of 21.21 Å, 20.60 Å, and 13.62 Å, respectively. The two AMPS-containing polymers had similar mean radii of gyration, both larger than that of PAM, indicating a greater spatial extent of the modeled chains at the hydrated kaolinite interface.

Nevertheless, the larger spatial span of CS-g-P(AM-AMPS) can increase the distance over which different segments contact suspended particles and is therefore consistent with a greater bridging potential. This interpretation agrees with chi-g-PAM/kaolinite studies in which a relatively extended conformation preserved accessible binding sites and strengthened bridging [24]. It is also consistent with chitosan-graft hydrogel work showing that long, flexible PAM side chains increased the accessibility of functional groups for noncovalent interactions [60].

**Figure 17:**
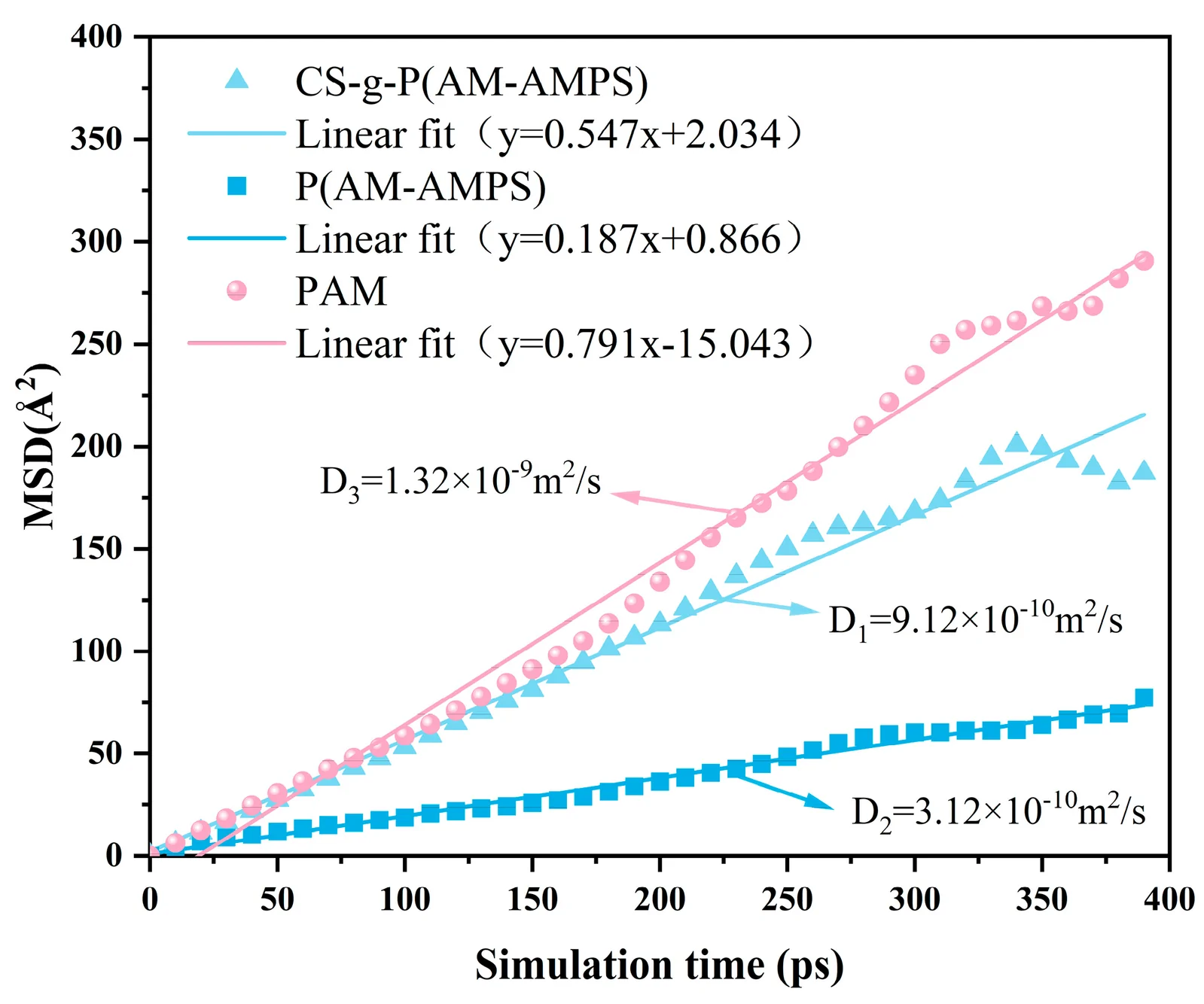
Mean square displacement and diffusion coefficient of polymers.

The three polymers exhibited increasing MSD values with different fitted slopes over the analyzed time interval, as shown in Figure 17. Among them, PAM shows the steepest MSD slope and the highest diffusion coefficient, followed by CS-g-P(AM-AMPS), whereas P(AM-AMPS) exhibits the lowest apparent mobility. Linear fitting of the MSD curves yields diffusion coefficients of 9.12 × 10^−10^ m^2^/s for CS-g-P(AM-AMPS), 1.32 × 10^−9^ m^2^/s for PAM, and 3.12 × 10^−10^ m^2^/s for P(AM-AMPS).

The MSD analysis shows that PAM has the highest apparent mobility, which is consistent with its linear structure and relatively low steric resistance. However, diffusion alone cannot explain final flocculation performance [39,61]. Although PAM diffuses more rapidly, its smaller radius of gyration limits long-range bridging. By contrast, CS-g-P(AM-AMPS) maintains a larger radius of gyration than PAM while retaining moderate apparent mobility. Taken together with the stronger interfacial affinity discussed in Section 3.3.1, these results indicate that the bridging ability of CS-g-P(AM-AMPS) arises from an adsorption-extension balance rather than from translational diffusion alone.

#### 3.3.3. Relative Concentration Distribution Analysis

**Figure 18:**
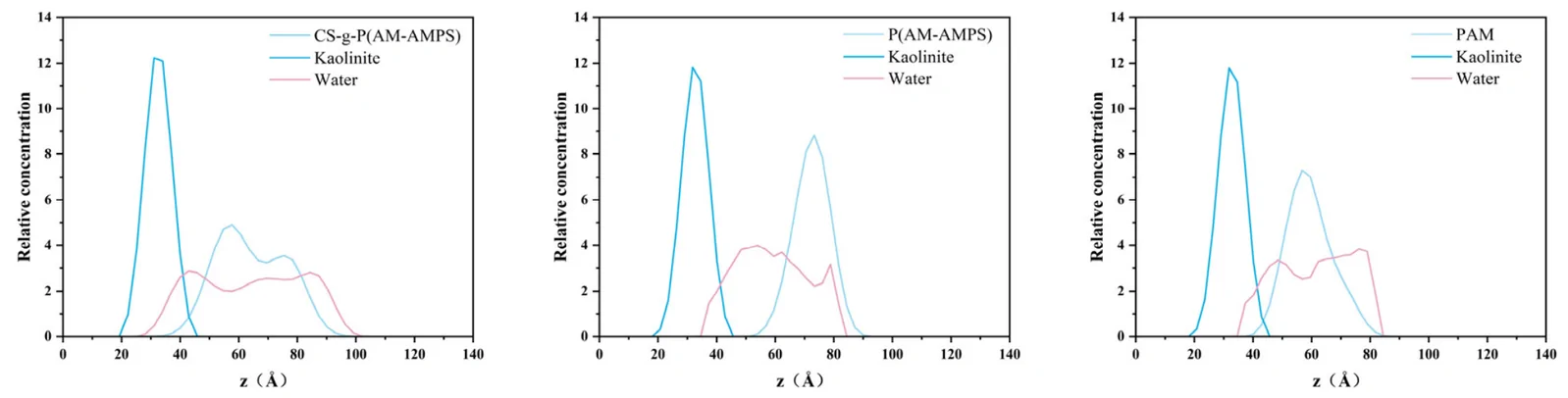
Interfacial concentration profiles of water and polymers along z-direction.

Figure 18 compares the spatial distributions of polymers and water along the direction normal to the kaolinite surface. PAM exhibits a pronounced, relatively narrow principal peak near the mineral surface, indicating that its chain segments are concentrated within a localized region of the hydrated interface.

For P(AM-AMPS), the principal polymer peak is located farther from the kaolinite surface than those of PAM and CS-g-P(AM-AMPS). Its relative concentration is lower immediately above the mineral layer, while its main enrichment occurs farther into the aqueous region. This profile indicates a distribution preferentially centered away from the immediate mineral interface, distinguishing the spatial arrangement of P(AM-AMPS) from that of the other two polymers.

CS-g-P(AM-AMPS) exhibits a lower principal peak than PAM but a broader distribution, with a shoulder extending toward higher z values. Polymer segments are distributed continuously from the near-surface region into the adjacent aqueous phase. Compared with PAM, the segment population is less confined to a narrow concentration maximum; compared with P(AM-AMPS), a greater part of the profile extends toward the mineral surface. The distinctive feature of the grafted polymer is therefore the coexistence of near-surface enrichment and a broader distribution of segments farther into solution.

The water profiles provide additional information about this interfacial arrangement. In the CS-g-P(AM-AMPS) system, the first water concentration maximum lies closer to the mineral layer than in the other two systems and overlaps the near-surface portion of the polymer profile. Chen et al. identified water molecules between the adsorbed cationic surfactant stearyl trimethyl ammonium chloride (STAC) and the hydroxylated kaolinite (001) surface from their concentration profiles [62]. In the present system, the overlapping water and polymer distributions identify a hydrated interfacial region occupied by CS-g-P(AM-AMPS). Together with its broader polymer profile, this spatial relationship supports a configuration in which near-surface segments coexist with interfacial water while other segments extend into the adjoining aqueous region.

#### 3.3.4. Hydrogen-Bond Analysis

**Figure 19:**
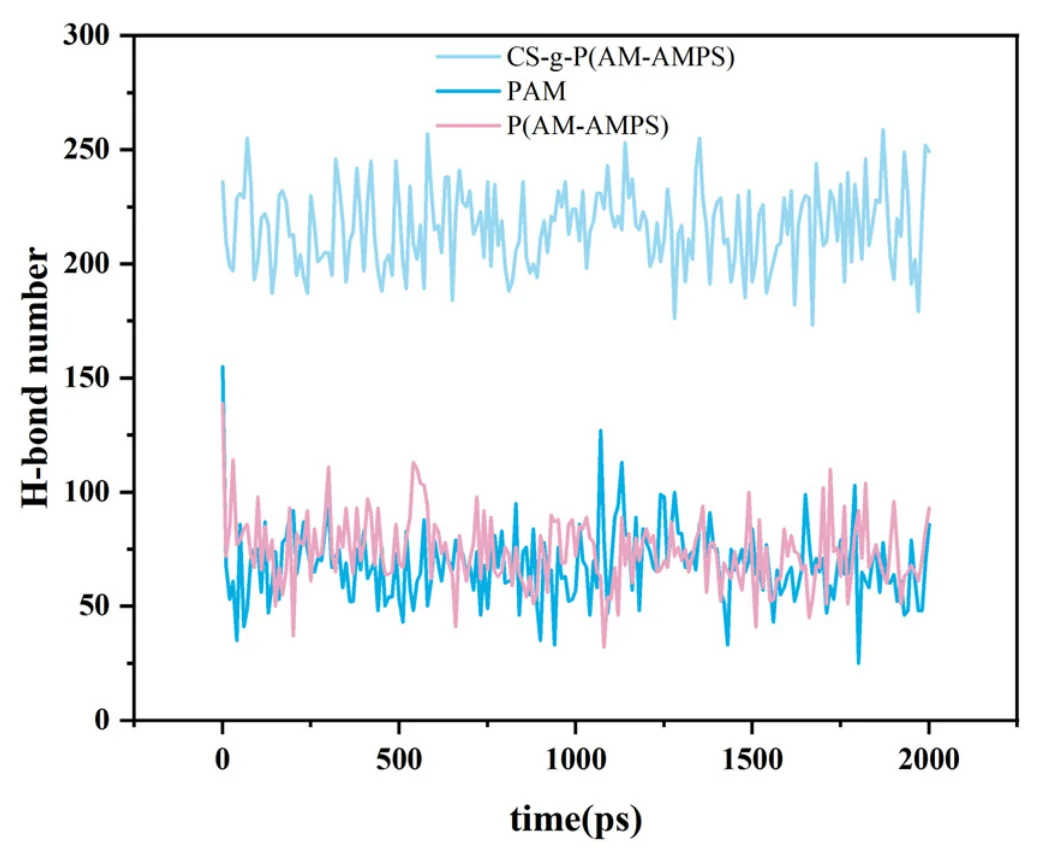
Time evolution of polymer–kaolinite interfacial hydrogen-bond populations.

Figure 19 and Table 4 show the time-dependent populations of hydrogen bonds between the three polymers and the kaolinite (001) surface over the 2000 ps trajectories. The average hydrogen-bond numbers were 216.00, 68.09, and 74.12 for CS-g-P(AM-AMPS), PAM, and P(AM-AMPS), respectively. The average population for CS-g-P(AM-AMPS) was therefore approximately 3.17 times that of PAM and 2.91 times that of P(AM-AMPS). The populations fluctuated with time, while the CS-g-P(AM-AMPS) curve remained clearly separated from those of both reference polymers. This sustained separation indicates that the grafted polymer maintained a substantially larger population of hydrogen-bonded surface contacts throughout the analyzed period.

The chitosan backbone introduces hydroxyl and amino groups alongside the amide and sulfonate groups of the AM/AMPS graft chains, providing additional potential sites for interfacial hydrogen bonding. Ge et al. identified four hydrogen bonds between a chitosan structural unit and the kaolinite (001) surface using DFT calculations, including two strong and two moderately strong contacts [63]. The strongest contact involved the chitosan nitrogen atom and a hydrogen atom of a surface hydroxyl group. Their findings identify a specific molecular route through which CS-derived groups can participate in surface hydrogen bonding. In the present hydrated polymer system, these additional groups provide a chemical basis for interpreting the larger total interfacial hydrogen-bond population of CS-g-P(AM-AMPS).

P(AM-AMPS) exhibited a slightly higher average hydrogen-bond number than PAM, and their time-dependent populations largely overlapped. Replacing part of the AM units with AMPS was therefore associated with a modest change in the total interfacial hydrogen-bond population. The CS-containing graft polymer showed a much more pronounced difference, sustaining a higher level of hydrogen-bond participation at the mineral surface. Combined with the concentration profiles in Figure 18, the hydrogen-bond analysis indicates that CS-g-P(AM-AMPS) maintains extensive surface contact while preserving an extended distribution toward the aqueous phase.

### 3.4. Flocculation Mechanism of Kaolinite

**Figure 20:**
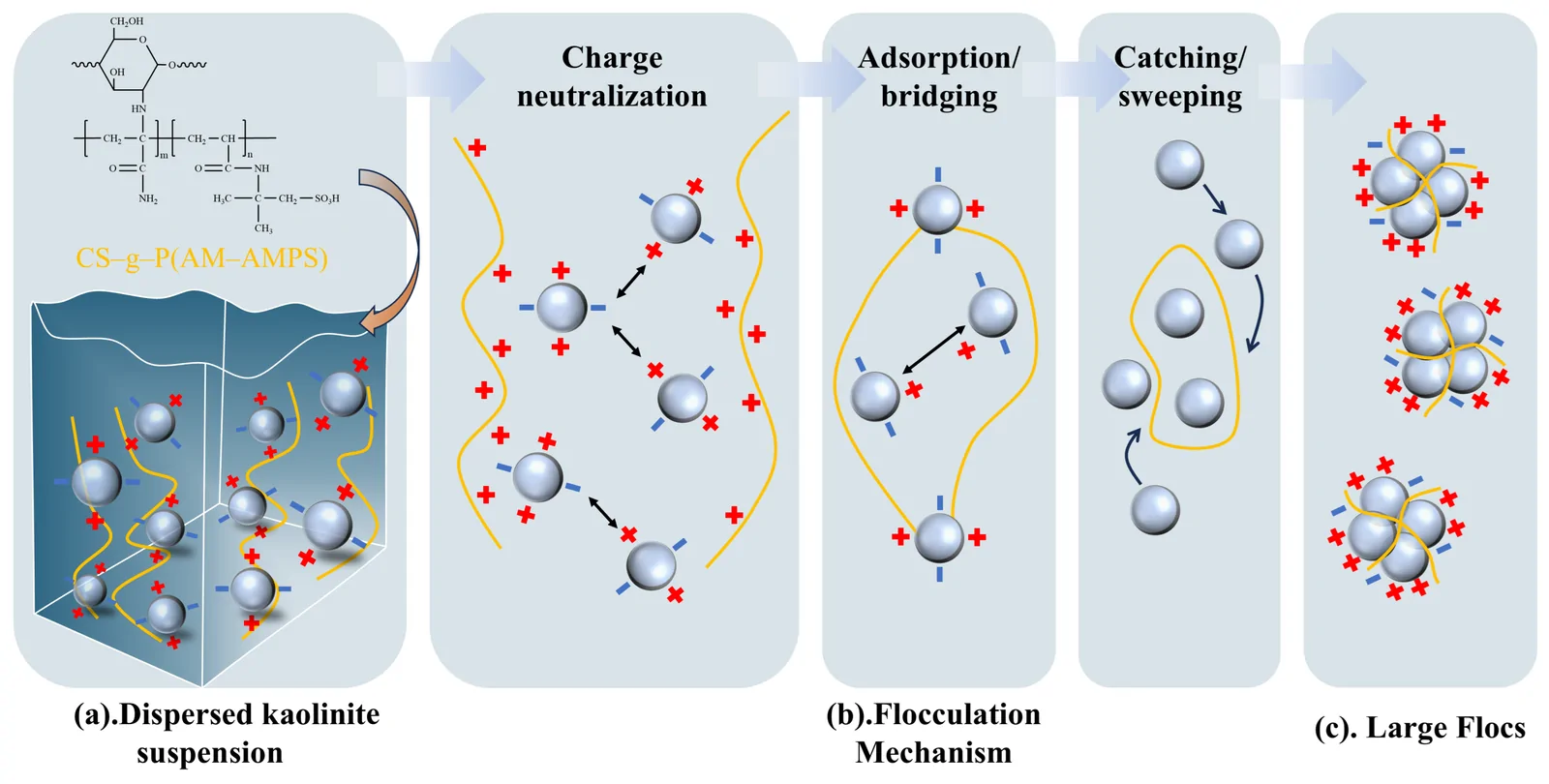
Flocculation mechanism of kaolinite suspended solids mediated by CS-g-P(AM-AMPS). Legend: light blue spheres = kaolinite; solid orange lines = CS-g-P(AM-AMPS); red + = positive charge; blue − = negative charge.

Figure 20 summarizes a proposed mechanism for kaolinite flocculation by CS-g-P(AM-AMPS). Under acidic conditions, protonated amino groups may assist association with negatively charged sites on kaolinite particles, while hydroxyl and amide groups provide opportunities for hydrogen bonding. The more negative polymer-surface interaction energy and larger interfacial hydrogen-bond population support extensive surface contact. Meanwhile, the radius of gyration and polymer concentration profile indicate a configuration extending into the aqueous phase. Such interfacial contact combined with chain extension can facilitate interparticle bridging, consistent with the larger flocs and higher turbidity removal observed experimentally.

P(AM-AMPS) produced larger median flocs and exhibited a slightly more negative polymer-surface interaction energy than PAM, yet achieved lower turbidity removal. The relative concentration profiles showed that P(AM-AMPS) was predominantly enriched farther from the mineral surface, whereas PAM exhibited a more localized concentration maximum near the interface. These distributions provide information on near-surface segment availability that complements the interaction-energy comparison. The larger median floc size and higher residual turbidity of P(AM-AMPS) also show that aggregate growth and suspension clarification followed different trends. These results suggest that PAM’s more localized interfacial distribution contributes to effective particle association and its higher turbidity removal.

## 4. Conclusions

CS-g-P(AM-AMPS) was prepared by ultrasound-assisted graft copolymerization and evaluated for the flocculation of model kaolinite suspensions, with P(AM-AMPS) and PAM as reference polymers. FTIR and XPS supported the expected functional-group composition of the product, while SEM revealed an irregular, porous dry-state morphology. Thermal analysis showed a higher second-stage decomposition peak temperature and greater residual mass than those of the reference polymers. Batch flocculation tests showed that CS-g-P(AM-AMPS) achieved higher final turbidity removal and produced larger kaolinite flocs than both reference polymers, although its removal efficiency at early settling times was lower than that of PAM.

MD simulations related these experimental differences to polymer-surface interactions and chain configuration at the hydrated kaolinite interface. CS-g-P(AM-AMPS) exhibited a more negative polymer-surface interaction energy and a larger interfacial hydrogen-bond population, while its radius of gyration and concentration profile indicated a relatively extended configuration spanning the interface and aqueous phase. These findings suggest that extensive surface contact combined with chain extension contributes to particle bridging and floc growth. The combined experimental and simulation results suggest that CS-g-P(AM-AMPS) has potential for removing kaolinite-rich suspended solids from water and wastewater through flocculation and sedimentation. Future work should assess floc density, settling behaviour, residual fines, floc strength and recovery after shear, and sludge volume to clarify separation performance and practical applicability.

## Figures and Tables

**Table 1:** Product composition analysis.

Elemental Composition (%)	CS-g-P(AM-AMPS)	P(AM-AMPS)	PAM
C	64.58	64.32	68.74
N	14.8	12.79	14.56
O	19.33	20.6	16.69
S	1.29	2.29	0

**Table 2:** Interaction energies between polymers and kaolinite (001).

Parameter	CS-g-P(AM-AMPS)	P(AM-AMPS)	PAM
Interaction energy (kcal/mol)	−314.674 ± 10.05	−266.396 ± 7.71	−258.935 ± 6.79

**Table 3:** Mean radii of gyration of the three polymers.

Parameter	CS-g-P(AM-AMPS)	P(AM-AMPS)	PAM
Mean *R*_g_ (Å)	21.21 ± 0.10	20.60 ± 0.09	13.62 ± 0.13

**Table 4:** Mean numbers of polymer–kaolinite hydrogen bonds.

Parameter	CS-g-P(AM-AMPS)	P(AM-AMPS)	PAM
Mean number of H-bonds	216.00 ± 5.31	74.12 ± 3.11	68.09 ± 2.82

## Data Availability

The raw data supporting the conclusions of this article will be made available by the authors on request.
